# A Comparison of Host Responses to Infection with Wild-Type Avian Influenza Viruses in Chickens and Tufted Ducks

**DOI:** 10.1128/spectrum.02586-22

**Published:** 2023-06-26

**Authors:** Mahmoud M. Naguib, Per Eriksson, Elinor Jax, Michelle Wille, Cecilia Lindskog, Caroline Bröjer, Janina Krambrich, Jonas Waldenström, Robert H. S. Kraus, Göran Larson, Åke Lundkvist, Björn Olsen, Josef D. Järhult, Patrik Ellström

**Affiliations:** a Zoonosis Science Center, Department of Medical Biochemistry and Microbiology, Uppsala University, Uppsala, Sweden; b Department of Migration, Max Planck Institute of Animal Behavior, Radolfzell, Germany; c Department of Biology, University of Konstanz, Konstanz, Germany; d Department of Immunology, Genetics and Pathology, Science for Life Laboratory, Uppsala University, Uppsala, Sweden; e Department of Pathology and Wildlife Diseases, National Veterinary Institute (SVA), Uppsala, Sweden; f Centre for Ecology and Evolution in Microbial Model Systems, Linnaeus University, Kalmar, Sweden; g Department of Laboratory Medicine, University of Gothenburg, Gothenburg, Sweden; h Laboratory of Clinical Chemistry, Sahlgrenska University Hospital, Gothenburg, Sweden; i Zoonosis Science Center, Department of Medical Sciences, Uppsala University, Uppsala, Sweden; University of Georgia

**Keywords:** influenza virus, gene expression, innate immunity, adaptive viral mutations

## Abstract

Cross-species transmission of influenza A virus (IAV) from wild waterfowl to poultry is the first step in a chain of events that can ultimately lead to exposure and infection of humans. Herein, we study the outcome of infection with eight different mallard-origin IAV subtypes in two different avian hosts: tufted ducks and chickens. We found that infection and shedding patterns as well as innate immune responses were highly dependent on viral subtypes, host species, and inoculation routes. For example, intraoesophageal inoculation, commonly used in mallard infection experiments, resulted in no infections in contrast to oculonasal inoculation, suggesting a difference in transmission routes. Despite H9N2 being endemic in chickens, inoculation of mallard-origin H9N2 failed to cause viable infection beyond 1 day postinfection in our study design. The innate immune responses were markedly different in chickens and tufted ducks, and despite the presence of retinoic acid-inducible gene-I (RIG-I) in tufted duck transcriptomes, it was neither up nor downregulated in response to infection. Overall, we have revealed the heterogeneity of infection patterns and responses in two markedly different avian hosts following a challenge with mallard-origin IAV. These virus-host interactions provide new insights into important aspects of interspecies transmission of IAV.

**IMPORTANCE** Our current findings highlight important aspects of IAV infection in birds that have implications for our understanding of its zoonotic ecology. In contrast to mallards where the intestinal tract is the main site of IAV replication, chickens and tufted ducks show limited or no signs of intestinal infection suggesting that the fecal-oral transmission route might not apply to all bird IAV host species. Our results indicate that mallard-origin IAVs undergo genetic changes upon introduction into new hosts, suggesting rapid adaptation to a new environment. However, similar to the mallard, chickens and tufted ducks show a limited immune response to infection with low pathogenic avian influenza viruses. These findings and future studies in different IAV hosts are important for our understanding of barriers to IAV transmission between species and ultimately from the wild reservoir to humans.

## INTRODUCTION

Key barriers to interspecies transmission of avian influenza virus (AIV) are molecular interactions between host and virus, including the adaptation of the virus to the new host, as well as the host’s innate and adaptive immune response to infection (1, 2). Avian influenza is a multihost virus, infecting an array of bird species through frequent cross-species transmission events. Despite this, we find differences in host competence and infection outcomes between avian species, likely driven by an array of factors (1, 3). For example, low pathogenic AIV replication in mallards (Anas platyrhynchos) (one of the most well-characterized AIV hosts) mainly occurs in the surface epithelium of the intestinal tract (4) whereas highly pathogenic AIVs preferentially replicate in the respiratory tissues (5). This is in contrast to domestic birds such as chickens, wherein the surface epithelium of the respiratory tract is the main site of replication for both AIV pathotypes (6–9). However, indirect evidence suggests that this is also the case in an array of wild bird species. A key example is diving ducks such as the tufted duck (Aythya fuligula) (10–12), and this is particularly evident when diving ducks are naturally infected with highly pathogenic avian influenza virus (HPAIV) (11). These differences in the site of infection have implications for transmission routes of the viruses and infection outcomes. For example, mallards are readily infected when AIV is experimentally administered via drinking water or by intraoesophaegal inoculation (13). Furthermore, mallards have shown variable morbidity and mortality based on the infecting strain (for example, H5 viruses of clade 2.3.4.4a versus 2.3.4.4b) (14), in contrast to tufted ducks (15, 16) and domestic birds (17), which are associated with high morbidity and mortality.

As with hosts, not all viruses are equal. Influenza A viruses are classified based on the virus surface proteins hemagglutinin (HA) and neuraminidase (NA) into subtypes H1 to H18 and N1 to N11, respectively. Subtypes H1 to H16 and N1 to N9 have been found in birds. Most of the 16 different AIV HA subtypes have been detected in the Eurasian wild mallard population, but there is substantial variation in the subtype frequency both within this commonly sampled species and between different avian taxa (18–20). In mallards, the most commonly detected HA subtypes include H1 to H6 and H10 (12, 21). This is in contrast to poultry, wherein an overrepresentation of H5, H7, and H9 is reported relative to other subtypes (19). The extraordinary subtype diversity found in mallards is not representative of all duck species, with few infections and subtypes reported in diving ducks, for example (12). The processes that dictate virus adaption between avian hosts are opaque, although several adaptive amino acid mutations have been identified in influenza virus proteins associated with the enhancement of virulence and transmission (6, 14, 22–24). There are certain differences between host responses in poultry versus in mallards: for example, domestic chickens lack the gene encoding the retinoic acid-inducible gene-I (RIG-I) protein (25). This difference has been proposed to be a key explanation for the more severe outcome of AIV infections in chickens compared to mallards, which possess this gene (25). However, although tufted ducks are also very vulnerable to infection with highly pathogenic AIV, they were recently shown to have an expressed analog of the RIG-I gene, suggesting that further studies are needed to better understand the role of this and other proteins in the avian host response to AIV (26).

Hence, if we are to understand the interspecies transmission of AIV in the wild bird reservoir, it is imperative to reveal factors influencing the virus-host arms race with an array of virus subtypes and host taxa in mind. As such, the aim of this study was to compare the outcomes of experimental AIV cross-species infections in two different avian hosts: (i) chicken, a well-studied animal model for AIV; and (ii) tufted duck, a nonmodel organism representative of the diving duck fauna, of which little is known about susceptibility to infection and host response. Critically, in both instances, we utilized a broad diversity of AIVs frequently isolated from mallards, the central reservoir for this virus. We assessed infection potential and host response to experimental infection with a broad array of mallard-origin low-pathogenic AIVs. We also monitored single nucleotide polymorphisms (SNPs) and intrahost single nucleotide variants (iSNVs) in virus genomes after infection.

## RESULTS

### The outcomes of experimental AIV infection differ between virus subtypes, host species, and routes of infection.

**FIG 1:**
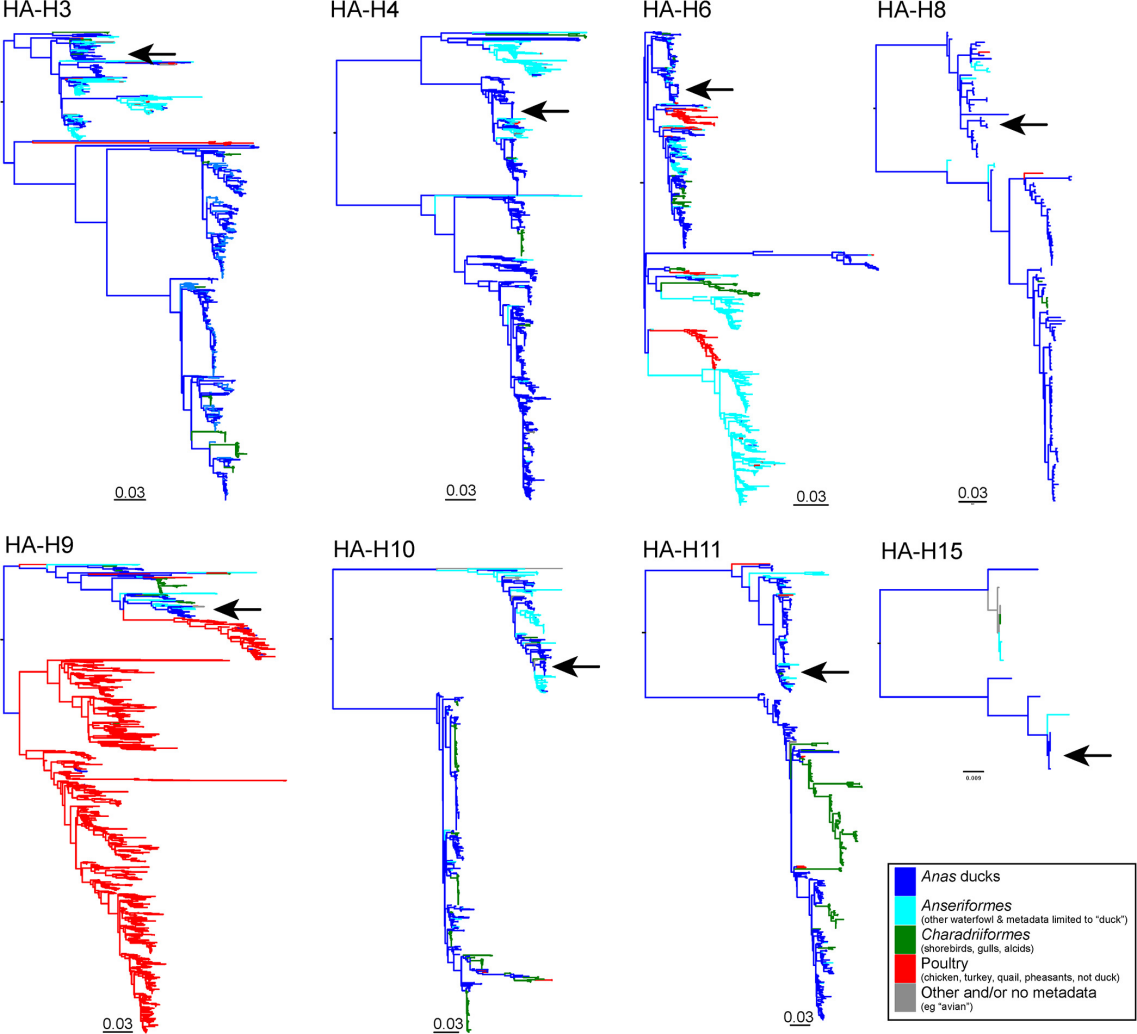
Low pathogenic avian influenza phylogeny. Maximum likelihood trees of the HA gene segments of influenza A virus subtypes used in this study. Sequences obtained from poultry including chickens, quails, turkeys, and pheasants are colored in red but do not include domestic ducks due to metadata limitations. Sequences obtained from *Anas* ducks are colored in blue. Sequences obtained from *Charadriiformes* (shorebirds, gulls, Alcidae) are colored in green. Sequences were obtained from *Anseriformes minus Anas* ducks, and all instances wherein metadata were missing (e.g., “duck”) are colored in gray. Scale bar indicates number of nucleotide substitutions per site.

To investigate how outcomes of AIV infection differ depending on host species and routes of infection, chickens and tufted ducks were infected with eight different subtypes of mallard-origin low pathogenic AIVs (Table S1 in the supplemental material) using two different routes of virus inoculation: oculonasal (ON) or intraesophageal (IE). Phylogenetic analyses of HA gene sequences from the viruses used in the experiments and all publicly available HA sequences from corresponding subtypes suggest that these viruses had circulated in mallards or other *Anas* species and did not represent recent crossovers from chickens or tufted ducks (Fig. 1). Furthermore, a close phylogenetic relationship was observed in all gene segments that were all located within the Eurasian lineage. However, the nonstructural (NS) gene segments of the H3, H4, and H8 viruses in our study are clustered with the NS allele B while the H6, H9, H10, H11, and H15 are clustered within NS allele-A (Fig. S2). In addition, *in silico* comparative analysis of amino acids at or near the HA receptor binding site was performed as described in reference 27 and displayed a predicted binding preference for avian-type receptors (Table S2) (22, 27).

**FIG 2:**
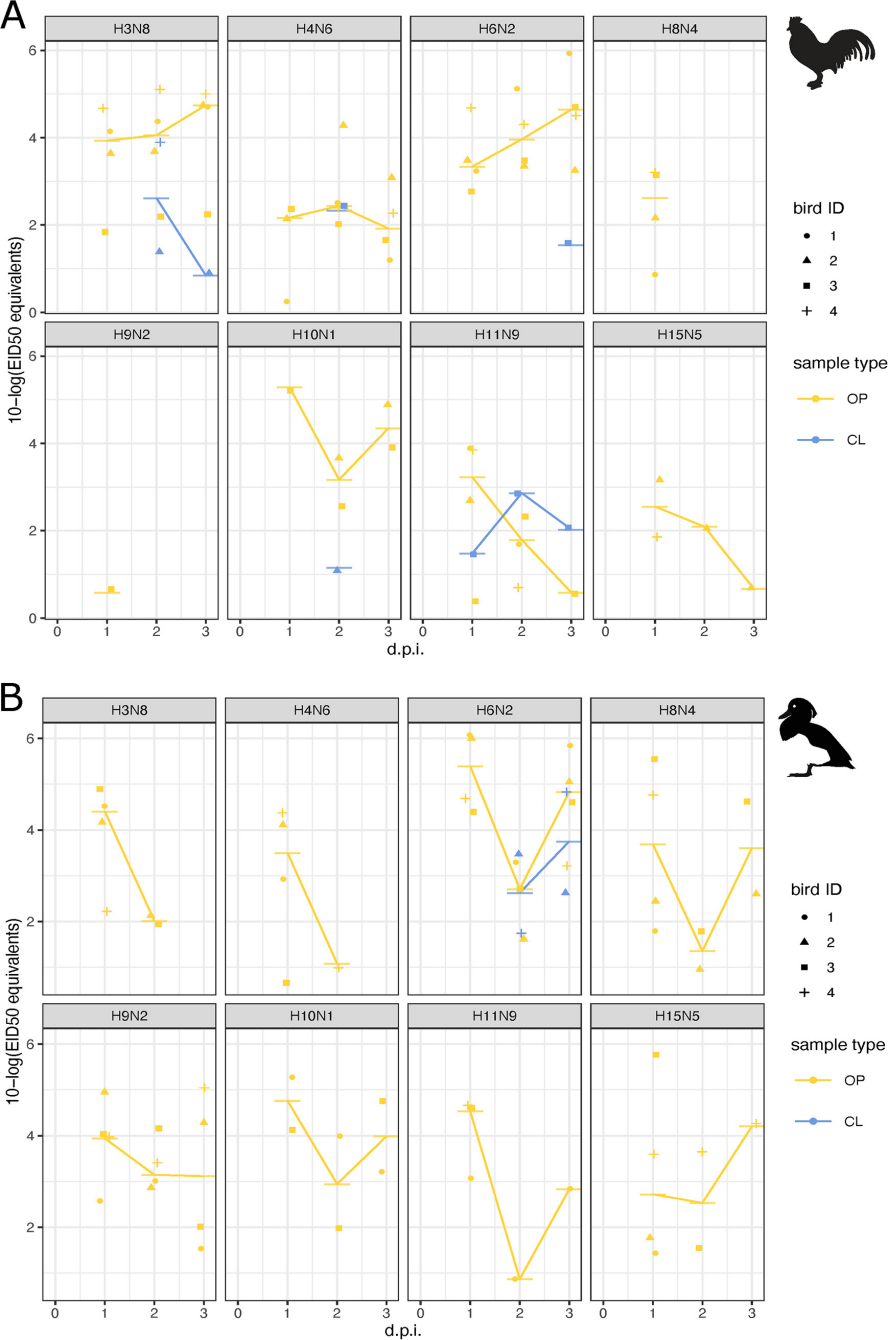
Viral load of mallard origin AIV subtypes in oculonasally inoculated chickens (A) and tufted (B) ducks. Four birds were inoculated with each virus, respectively. Points represent positive samples and horizontal bars their respective median values and are colored by sample type: oropharyngeal (OP; yellow) and cloacal (CL; blue) swabs. Birds 1 to 4 are indicated with corresponding shapes. The trajectory lines are based on the daily median titer per sample type. Negative samples are not shown. The *y* axis corresponds to log_10_ 50% egg infective dose equivalents that are inferred from *C*_q_ values based on the relationship reported in Fig. S1.

**FIG 3:**
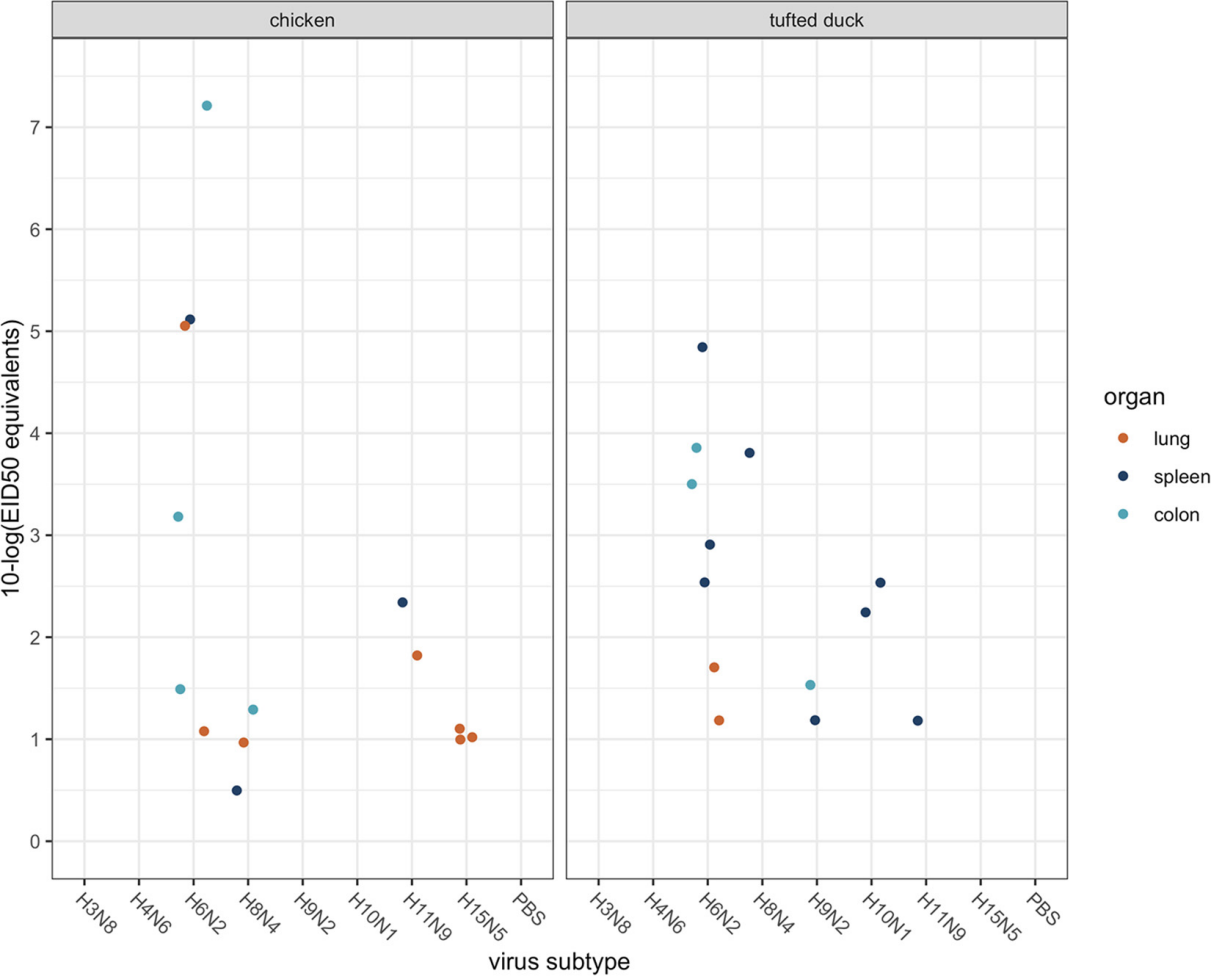
Strain-specific AIV positive organ samples. Scatterplot of obtained positive organ samples at 3 days postinfection from ON inoculated chickens and tufted ducks. Each point illustrates one positive sample from one individual. Four individuals were inoculated with each virus, respectively. The points are colored based on organ type; orange, lung; dark blue, spleen, and light blue, colon. Log_10_ 50% egg infective dose equivalents versus inoculum.

We found a substantial difference in infection outcomes in both chickens and tufted ducks depending on the route of inoculation. All chickens and tufted ducks inoculated intraesophageally were negative in both oropharyngeal and cloacal swab samples by reverse transcriptase-quantitative real-time PCR (RT-qPCR) with only two exceptions: one out of four tufted ducks inoculated with H8N4 showed positive AIV RNA (2.54 10-log 50% egg infective dose [EID_50_] equivalents) at 1 day postinfection (1 dpi) and one out of four tufted ducks inoculated with H11N9 (0.79 10-log EID_50_ equivalents) at 2 dpi. In contrast, oculonasal inoculation yielded positive oropharyngeal samples for all viruses in both host species at 1 dpi (Fig. 2A). In oculonasally inoculated chickens, virus shedding was detected in the oropharyngeal swabs on all days in all four chickens inoculated with H3N8, H4N6, and H6N2 viruses. In chickens inoculated with H10N1, H11N9, and H15N5, oropharyngeal shedding continued until 3 dpi in two (H10), one (H11), and one (H15) birds, respectively (Fig. 2A). The H8N4 virus was detected in oropharyngeal samples only at 1 dpi although this virus was detected in the lung, spleen, and colon at 3 dpi (Fig. 3). Only one out of four chickens inoculated with the H9N2 virus had any indication of viral shedding (0.58 10-log EID_50_ equivalents), and this was limited to the oropharyngeal sample at 1 dpi. Given the high burden of H9N2 in poultry, this is interesting. However, phylogenetically the virus used in this study falls into a clade dominated by wild bird sequences from Europe, which was distantly related to clades dominated by H9N2 viruses found in Asian poultry (Fig. 1). Beyond oropharyngeal and cloacal samples, the H6N2 virus showed the most widespread tissue related viral replication in chickens, with AIV RNA detected in the colon of three birds, lungs in two birds, and spleen in one bird at 3 dpi with virus titer up to 7.27 10-log EID_50_ equivalents. For the other subtypes, viral replication in internal organs was detected to various extents (Fig. 3).

In the oculonasally inoculated tufted ducks, all birds in all groups shed AIV RNA in oropharyngeal swabs at 1 dpi, with the exception of the H10N1 and H11N9 virus inoculated groups, where only two and three birds, respectively, were positive (Fig. 2B). The H6N2 and H9N2 groups showed virus RNA in the oropharyngeal swabs from all tufted ducks until 3 dpi and from two birds in the cloacal swabs (Fig. 2B). Virus RNA was detected at 3 dpi in different tissues (as shown in Fig. S3). No signs of disease and no virus were detected in any of the collected swab samples in the control groups.

**FIG 4:**
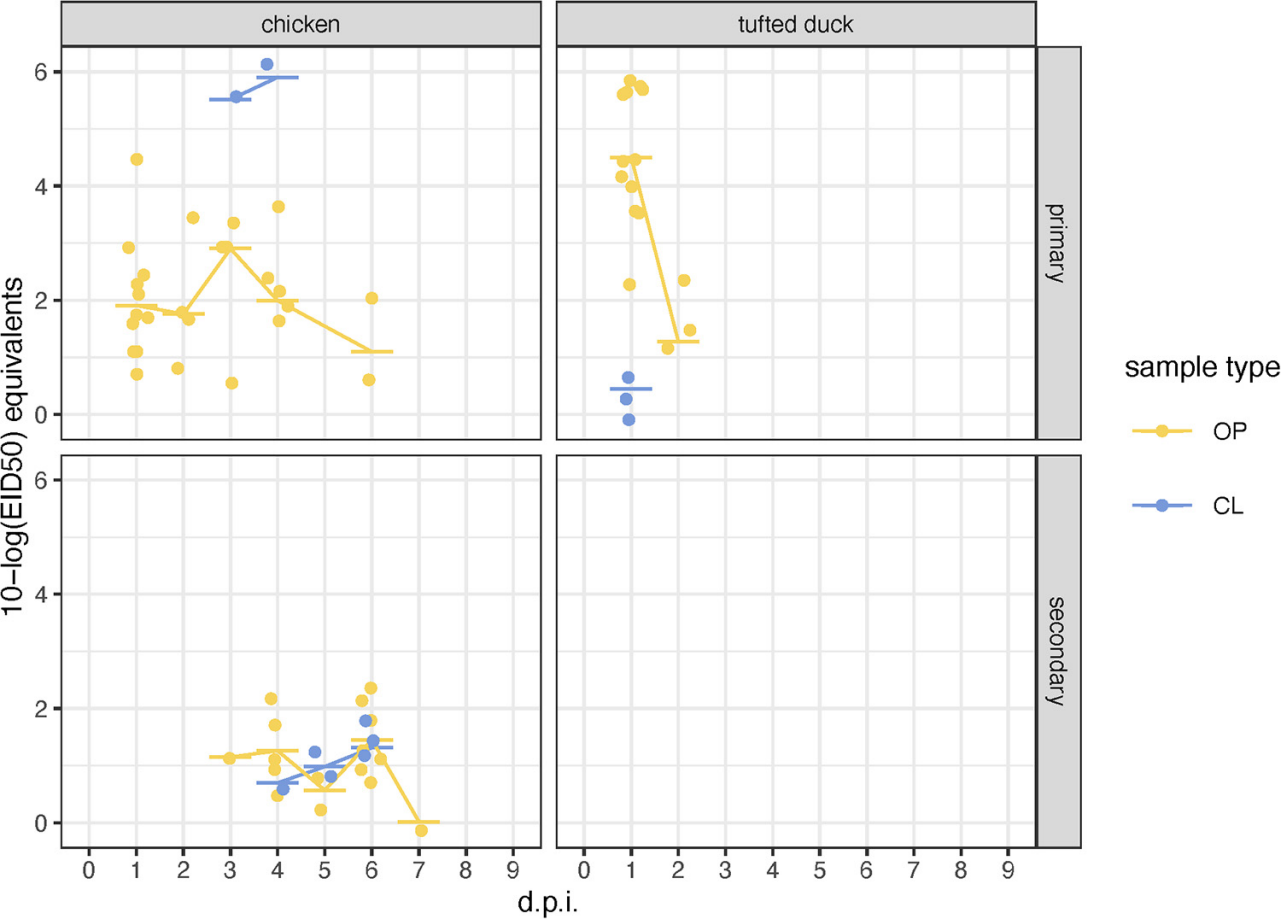
Virus shedding collected from oropharyngeal (OP) and cloacal (CL) swabs followed H8N4 virus oculonasal inoculation. Points represent positive samples and horizontal bars their respective median values and are colored by sample type OP (yellow) and CL (blue) swabs. The trajectory lines are based on the daily median titer per sample type. Negative samples are not shown. The *y* axis corresponds to log_10_ 50% egg infective dose equivalents that are inferred from *C*_q_ values based on the relationship reported in Fig. S1.

In the contact experiment with H8N4, inoculated chickens were positive for influenza virus RNA in oropharyngeal swabs from 1 dpi and up to 6 dpi although virus titers were generally low (Fig. 4). The number of birds shedding virus in oropharynx decreased with time and at 6 dpi only two birds had positive swabs. Cloacal shedding of the virus was detected in one bird at 3 and 4 dpi. In secondary-introduced (IAV naive) chickens, viral shedding was detected in oropharyngeal swabs in up to six birds at 6 dpi (4 days after introduction). The first positive bird was detected at 3 dpi, and the last bird shed the virus until 7 dpi. Cloacal shedding was detected in one bird at 4 dpi and in three birds at 6 dpi. Of the inoculated tufted ducks, all birds had positive oropharyngeal swabs at 1 dpi and only three birds at 2 dpi, and the virus was detected in three cloacal swabs from three birds at 1 dpi. No virus was detected in the oropharyngeal or cloacal swab samples collected from the secondary-induced tufted ducks (Fig. 4).

Taken together, viral shedding differed depending on the AIV subtype, host species, and routes of inoculation. The intraeosophageal inoculation route produced no virus infections in either tufted ducks or chickens. Although we did not perform a direct comparison by inoculating mallards with the same subtypes in this study, this outcome is in contrast to previously reported results from mallards, where intraesophageal inoculation resulted in cloacal shedding regardless of viral subtype in multiple studies (13, 28–30). This suggests that the site of replication might differ between bird species, even within the *Anatidae* family.

### A diversity in progeny viruses following experimental infection.

Putative viral genetic changes during virus infection of chickens and tufted ducks were investigated in each individual swab and tissue sample with virus EID_50_ equivalents high enough to allow for deep sequencing and compared to the inoculum (Table S3). No deletions or insertions were recorded in any of the samples. However, synonymous and nonsynonymous variants were detected (Table S3). SNPs/iSNV conferring previously described amino acid variations associated with host specificity were detected in the nucleoprotein (NP) protein (N319K) of the tufted duck/H6 spleen (99.7% in the sample, 0% in the inoculum) and in the HA (N133D) of the chicken/H3 at 3 dpi (1.4% in the sample and 1.1% in the inoculum) (Table S3) (31–33). Furthermore, iSNVs/SNP involved in antibody recognition sites were observed in the HA: F120L and T167A in the chicken/H3 (3.8% and 1.3% in the sample and 0% in the inoculum for both); M209T in chicken/H6 (1.8% in the sample and 0.1% in the inoculum); Y141C and H184R in tufted duck/H6 (1.3% and 1.8% in the sample and 0% and 0.1% in the inoculum); I318V in tufted duck/H8 (76.3% in the sample and 14.3% in the inoculum); and K156E in chicken/H10 and S128P in tufted duck/H10-infected groups (2.5% in the samples and 0% in the inoculum) (Table S3). A V27I amino acid substitutional change in the M2 protein, previously described in association with amantadine resistance (34), was observed in the virus retrieved from tufted duck/H6 colon (51.8% in the sample and 0.1% in the inoculum). In addition, L43F was found in the M2 in both chicken/H6 lung and spleen (84.9% and 99.5% in the sample and 0% in both inoculums) as well as tufted duck/H6 colon and spleen (34.9% and 99.6% in the sample and 0% in both inoculums). Furthermore, SNPs/iSNV of unknown significance were observed in different genes as shown in Table S3.

### Transcriptomics demonstrates differential expression of genes involved in innate immunity and glycosylation in chickens and tufted ducks.

To understand the host response of chickens and tufted ducks after a challenge with mallard-origin AIVs, we utilized RNA sequencing (RNA-seq) to disentangle the differential gene expression patterns of the hosts. We selected 216 tissue samples (obtained at 3 dpi) comprising the lung and colon, as they are important sites of infection, and the spleen as it is a primary lymphoid organ. Out of the 216 samples sequenced, 207 were of high quality with an average of 3,311,801 ± 740,763 (mean ± SD) reads per sample. The remaining nine samples were excluded due to low sequencing depth and/or quality.

First, we identified significant differentially expressed genes (DEGs) in each tissue (colon, lung, and spleen) for chickens and tufted ducks for each of the eight AIV subtypes. In chickens, the number of DEGs ranged from 11 to 2,949 in the colon, 34 to 3,225 in the lung, and 6 to 3,046 in the spleen and in tufted ducks from 49 to 597 in the colon, 7 to 416 in the lung, and 92 to 560 in the spleen of birds treated with the different AIV subtypes, see (Fig. S3A to B and S4A to B). A marked difference in response to virus challenge in chickens between H3N8 or H4N6 and the other subtypes tested was observed in that these subtypes had markedly fewer DEGs than the rest. Fewer DEGs were identified in the tufted duck organs (mean 248 DEGs/organ/virus SD ± 167), and in contrast to what was observed in chickens, H3N8 was the subtype eliciting the highest number of DEGs. The overlap of DEGs was evaluated between groups infected with the different AIV subtypes within the same species, as well as groups infected with the same subtype between the two species. The proportion of DEGs in response to infection that was unique to each AIV subtype was calculated. In chickens, it ranged from 3 to 32% in the colon, 4 to 24% in the lung, and 0 to 31% in the spleen across different subtypes (Fig. S5A to C). Chickens inoculated with H3N8 had the highest percentage of unique DEGs (32% in colon, 23% in lung, and 31% in spleen). The number of unique DEGs detected in a single treatment group in tufted ducks ranged from 6 to 51% in the colon, 0 to 32% in the lung, and 10 to 84% in the spleen (Fig. S5D to F). As in chickens, H3N8-infected tufted ducks generally displayed the highest percentage of unique DEGs (51% in colon, 31% in lung, and 42% in spleen). For all viruses except H3N8 and H4N6, the numbers of DEGs in response to infection were lower in tufted ducks than in chickens. The overlap of orthologous DEGs between the two species was low to moderate, with H3N8 and H4N6 having a smaller overlap of DEGs between the two species than the remaining virus subtypes (Fig. S6 to S8). Gene ontology and pathway analysis of chicken lung, spleen, and colon samples mainly identified terms associated with nuclear and organelle lumen and nucleotide binding (data not shown), i.e., terms/pathways mainly associated with generic cellular functions. The DEGs unique to the H3N8 treatment groups in chicken and tufted duck were involved in a wide range of biological processes, including cellular process (GO:0009987), metabolic process (GO:0008152), biological regulation (GO:0065007), localization (GO:0051179), and response to stimulus (GO:0050896). However, few of the DEGs in general or unique to the H3N8 treatment group were involved in the immune system process (GO:0002376), albeit with some exceptions (chicken, gamma-glutamyltransferase 2; tufted duck, colony-stimulating factor 1 receptor and interleukin 16).

**FIG 5:**
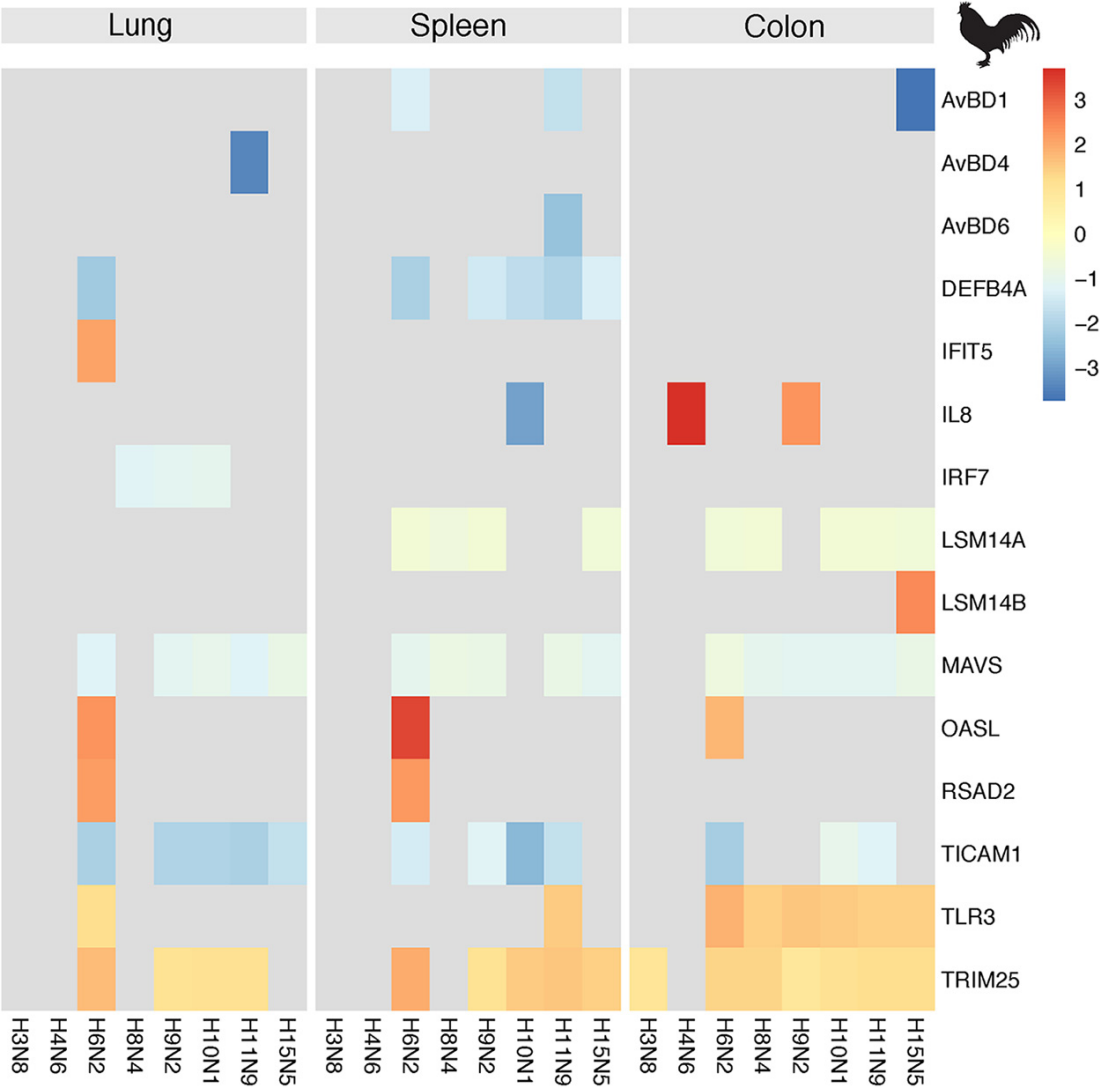
Selected significant differentially expressed genes (DEGs) associated with innate immunity in chickens. Genes in the proinflammatory or RIG-I-like receptor (RLR) signaling pathways as well as β-defensin genes with described functions in birds were selected (Table S4) based on current literature. The color indicates the log_2_-fold change of significant (adjusted *P* value <0.05 and a threshold of ≥10%-fold change) DEGs relative to control birds. Gray indicates genes that were not significantly differentially expressed. Data from lung samples are shown on the left, spleen in the center, and colon on the right. Tissues were obtained at 3 dpi.

To assess the innate immune response to the AIVs used in this study, we specifically studied genes known to be involved in interferon and proinflammatory pathways as well as β-defensins in chickens and ducks at 3 dpi (3, 35). The full list of genes included in this analysis is found in Table S4. For chickens, there was good coherence in the expression level of such genes between the replicate birds within the negative-control group and within the groups infected with each of the eight viruses as shown in Fig. S9A to C displaying gene expression level for each individual bird. In general, up- or downregulation of such genes was weak or absent in both chickens and in tufted ducks. In chickens, a few genes related to interferon signaling were generally affected in response to infection; in the colon, *TLR 3* was weakly upregulated for all subtypes except for H3N8 and H4N6 (Fig. 5 and Fig. S9 and Table S5). However, the adaptor molecule *TICAM1/TRIF* downstream of TLR 3 was generally downregulated or unaffected in the corresponding colon samples as well as in samples from the lung and spleen (Fig. 5 and Fig. S9). The ubiquitine ligase gene *TRIM25* was weakly upregulated in response to most IAV subtypes in the colon, spleen, and lung of chickens. Among the β-defensin genes analyzed, significant changes in response to AIV infection were mainly detected in the spleen (and to some extent in the lung) of chickens, where *AvBD1*, *4*, and *6* as well as *DEFB4A* were all downregulated at 3 dpi. In infected chickens, the H6N2 subtype stood out in evoking the most consistent responses in genes related to interferon (IFN) signaling (Fig. 5 and Table S4). From Fig. S9, showing the gene expression level in each of the replicate birds, it is evident that this effect was mainly driven by bird H6N2 C1, which showed high virus loads in all three tissues. Mueller et al. (26) recently showed that the RNA-sensing protein RIG-I is encoded and expressed in the tufted duck. However, the only significant innate immunity-associated DEGs recorded in tufted ducks were Nitric oxide synthase 2 (NOS2) was highly upregulated in the lungs of birds infected with H4N6 and H6N2 (log_2_-fold change, 3.3 and 3.4, respectively), and *TRIM25* was weakly upregulated in the colon of H6N2-infected birds (log_2_-fold change 0.8).

**FIG 6:**
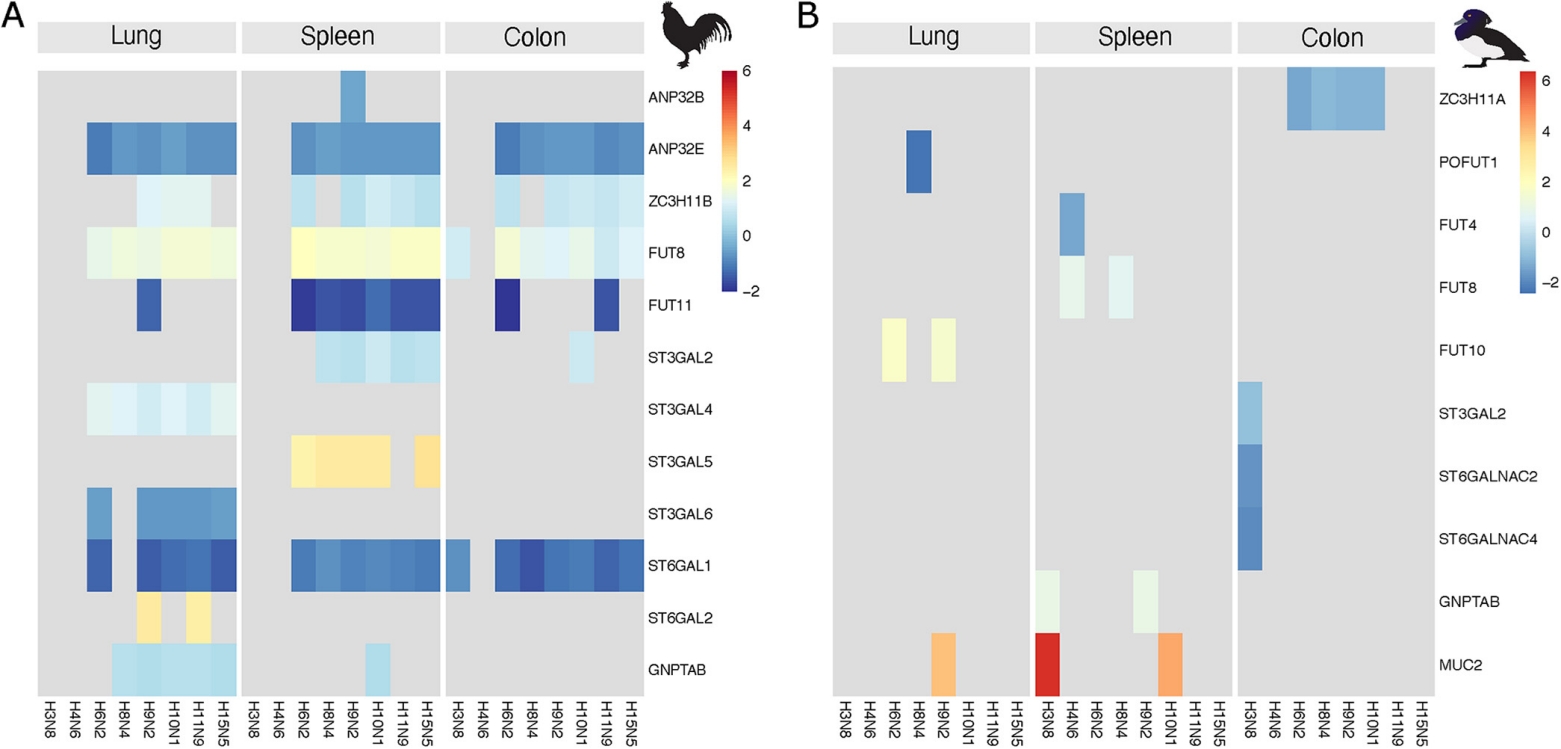
Selected significant DEGs associated with glycosylation and transcription in chickens and tufted ducks. Genes with described associations to glycosylation or transcription in birds (Table S2) were selected based on current literature. The color indicates the log_2_-fold change of significant (adjusted *P* value <0.05 and a threshold of ≥10%-fold change) DEGs relative to control birds. Gray indicates genes that were not significantly differentially expressed. Data from chicken (A) and tufted duck (B) lung samples are shown in on the left, spleen in the center, and colon on the right. Tissues were obtained at 3 dpi.

Other genes of note, are the ANP32 family, the proteins of which are serving as cofactors for the virus polymerase during transcription. In chickens, ANP32E was weakly downregulated in all tissues in response to infection with all subtypes except for H3N8 and H4N6 (Fig. 6A and Table S5). The zinc finger protein ZC3H11A has been suggested to affect the replication efficiency of several nuclear replicating RNA viruses including IAV (36). In our experiments in chickens, ZC3H11B was weakly upregulated for several of the IAV subtypes in all tissues (Fig. 6A and Fig. S10 and Table S5 and S6), whereas in tufted ducks ZC3H11A was found slightly downregulated in the colon for some of the IAV subtypes (Fig. 6B and Fig. S11 and Table S5 and S6).

Glycosyltransferases related to AIV receptor synthesis were differentially expressed in chickens at 3 dpi. Of note was that sialyl transferases adding sialic acid α2,3-linked to Gal were generally weakly upregulated in response to infection for most viruses (*ST3GAL2* in spleen, *ST3GAL4* in lung, *ST3GAL5* in spleen) whereas *ST6GAL1* encoding a sialyl transferase adding sialic acid α2,6-linked was weakly downregulated in all chicken tissues for most viruses, except H3N8 and H4N6. Exceptions to this rule were *ST3GAL6*, which was downregulated in the lungs, and *ST6GAL2*, which was generally unaffected but strongly upregulated in the lungs of chickens infected with H9N2 and H11N9 (Fig. 6A and Fig. S10 and Table S6). A similar pattern could not be observed in tufted ducks where the transferase expression was rather unaffected by the viral challenges (Fig. 6B and Fig. S11 and Table S6). The *FUT8* gene, coding for the α1,6-fucosyltransferase responsible for core fucosylation of *N*-glycans, was generally upregulated in all chicken tissues for most viruses, again except for H3N8 and H4N6, whereas *FUT11*, coding for an α1,3-fucosyltransferase, was downregulated, predominantly in the chicken spleen (Fig. 6A and Fig. S10 and Table S6). In tufted ducks, these genes were generally unaffected except for weak downregulation of *FUT8* in the spleen in response to H4N6 and H8N4 (Fig. 6B and Fig. S11 and Table S6).

## DISCUSSION

Molecular barriers for transmission of influenza A viruses between birds and humans can be classified into (i) host-related factors, e.g., species-specific physiological, biochemical, and immunological differences; and (ii) virus-related factors, e.g., subtypes, sublineage-specific genetic markers associated with adaptation to a specific host as well as receptor and organ tropism (37). Mallards are regarded as a key reservoir for AIV, as they carry almost all known AIV subtypes (38, 39) and show limited signs of disease even when infected with many highly pathogenic AIV (HPAIV) (40). In contrast, HPAIV causes significant morbidity and mortality in other bird species including related ducks, such as Eurasian wigeons (15) and tufted ducks (15, 41), as well as in chickens (42). Using experimental infection of chickens and tufted ducks with influenza A viruses of mallard origin, we assessed the outcome of infection, host response, and viral genetic changes during virus infection. The results reveal a number of features that are important for our understanding of the infection dynamics of AIV in different bird hosts.

First, we found that low pathogenic AIVs preferentially replicated in the respiratory tract of tufted ducks and chickens. Although AIV replication can occur in the respiratory tract of mallards, replication of low pathogenic AIV preferentially occurs in the surface epithelium of the gastrointestinal tract, and transmission is considered to mainly occur via the fecal-oral route (4, 5, 39) The unsuccessful infection of tufted ducks via the intraesophageal route was thus unexpected as both mallards and tufted ducks belong to the *Anatidae* family (43), and it is long assumed that all duck species share the same mode of AIV transmission. Of note is that previous studies on experimental infection in mallards with this inoculation method, using several different AIV subtypes, have resulted in a successful infection in close to 100% of the birds (13, 28–30). On the other hand, AIV prevalence can differ dramatically between species and tribes within the *Anatidae* family (44). Additionally, studies using virus histochemistry found more virus staining in the trachea, rather than in the colon for several nonmallard species, including the tufted duck (10), suggesting differences in tissue tropism and receptor expression across this avian family. Although we cannot exclude that positive oropharyngeal samples at 1 dpi might reflect remains of the inoculum, similar findings were reported in a recent study in chickens (6), and true infection was supported by positive samples from internal organs of the same bird individuals at 3 dpi for some of the viruses in our study (Fig. 3). That diving ducks have infection profiles more consistent with chickens than mallards is a crucial finding. Taken together, our findings suggest substantial heterogeneity in the host capacity of waterfowl species and that such differences in AIV host capacity and transmission between bird species are due to differences in AIV tropism and infectivity.

Second, we found substantial heterogeneity in the outcome of experimental infection across the eight different AIV subtypes tested. Notably, H6N2 was detected at the highest virus loads both in oropharyngeal swabs and in tissue samples from both chickens and tufted ducks. This is perhaps not surprising as this virus subtype is very common in wild mallards (38, 45, 46) and has been demonstrated to infect gallinaceous birds, including pheasants and quails (47) as well as chickens (19). Our findings are corroborated by previous studies, similarly illustrating that when wild bird origin AIV is inoculated into chickens, the outcome of infection varies based on virus subtype, but there can also be great variations in virus titers in, e.g., oropharyngeal swabs within a group infected with the same virus (6, 48). In general, fecal shedding was low or absent for most subtypes. This is in direct contrast to studies done using mallards, wherein fecal shedding is high, as early as day 1. A limitation of the study is that the duration of the infection experiments was only 3 days. Hence, it cannot be excluded that low-level fecal shedding in our study was an effect of the rather short duration of the experiments, such that fecal shedding may be delayed in chickens and tufted ducks. However, in our long experiments where H8N4 inoculated birds were followed up to 9 days, cloacal shedding was detected only at 1 dpi in tufted ducks and 2 to 3 dpi in chickens, well in line with the very rapid and short-lived fecal shedding seen in mallards. Although fecal shedding in the secondary introduced chickens was somewhat delayed (2 to 4 days after introduction), the duration of shedding was transient, suggesting that the risk that we missed cloacal shedding in the short experiments is likely to be low. The unsuccessful infection of chickens with the H9N2 subtype was interesting as low pathogenic AIV H9N2 is endemic in chickens in many countries (49). The fact that the HA gene of our H9N2 isolate was phylogenetically distinct from poultry endemic H9N2 isolates indicates the importance of sublineages within the same AIV subtype that seems to be adapted to different host species. We aimed to better understand if there was any adaptation occurring following the inoculation of mallard-origin AIV into other species through the detection of SNP/iSNVs. Several SNPs and iSNVs were identified in swab and organ samples collected from chickens infected with H3N8, H6N2, and H10N1 and tufted ducks infected with H6N2, H8N4, H9N2, and H10N1 (Table S3). It is possible that at least some of these are associated with infection of a new host species. Among the amino acid substitutions found, the NP (N319K) and HA (N133D) have previously been described to enhance the interaction with the host factor importin-α conferring increased virus replication and a human-like receptor binding profile, respectively (31–33). However, the vast majority of SNP/iSNVs have not yet been associated with any viral changes. Hence, further studies are needed to confirm that SNP/iSNVs occurred in adaptation to a new host species and to elucidate the role of these viral amino acid substitutions.

Third, transcriptomic analysis showed that the host response to virus challenge differed between chickens and tufted ducks. For instance, AIVs H3N8 and H4N6, viral subtypes, which are very common in the mallard reservoir, generated the least number of DEGs in chickens, in contrast to tufted ducks wherein they yielded the greatest number of DEGs (Fig. S4). Gene ontology and pathway analysis of DEGs in chicken and tufted duck tissue samples identified only very few DEGs related to the Immune system process (GO:0002376). This suggests that the chickens and tufted ducks did not exhibit a strong immune response to the AIVs tested, in line with the low virus replication and what has been seen in experimental infections with low pathogenic AIV in mallards (3). The H6N2 subtype gave the most prominent upregulation of IFN-related genes in all chicken samples, consistent with its replication in the lungs, spleens, and colons (Fig. 5), and this response was in particular driven by one individual with high virus loads in these organs. However, a similar response to this subtype was not detected in tufted ducks, despite the presence of the *RIG-I* gene in this species (26). It should be emphasized that the samples used for the transcriptomic analysis in our experiments were taken at 3 dpi whereas in other studies, the strongest innate immune responses to AIV infection in chickens and ducks take place at 1 to 2 dpi (6, 50, 51). Furthermore, virus replication was generally low and varied between individual birds within each group. A comparison of the outcome of infection between highly pathogenic AIV H5N1 and low pathogenic AIV H9N2 in chickens showed markedly reduced gene expression for the low pathogenic compared to the highly pathogenic AIV (52). Similarly, infection in Pekin ducks with highly pathogenic AIV H5N1 and low pathogenic AIV H5N2 showed a lower response to the low pathogenic AIV with a peak at 2 dpi rather than at 3 dpi for the highly pathogenic AIV (53). The upregulation of *ZC3H11B* in chickens is interesting and merits further studies as the A ortholog was described to be involved in the nuclear export of mRNA in humans and that knocking this gene out in HeLa cells resulted in reduced growth of the IAV strain H1N1 A/WSN/33 (36). Taken together, only minor changes in gene expression were identified in virus-challenged birds, well in line with earlier reports of low pathogenic AIV only inducing mild responses in birds (6, 50–53). This was likely also an effect of the generally low replication of the viruses in these birds. Higher virus replication would likely have caused stronger immune responses as illustrated by the H6N2 experiments, where the stronger host response in one individual correlated with high virus titers in the organs. However, although we could not detect the virus in all organs used for transcriptomic analysis, we cannot exclude that virus was present in these organs at earlier time points during infection. It should be noted that in all transcriptomics experiments, we compared the gene expression in infected birds to that in noninfected control birds to calculate the fold change of each DEG (displayed in Fig. 5 and 6) (see Materials and Methods). As a complement, we also included the normalized expression level of each gene for each individual bird (displayed in Fig. S9, S10, and S11). Both in terms of the number of significant DEGs identified and particular up-/downregulated DEGs, clear differences could be observed in the host response to various virus subtype challenges between chickens and tufted ducks, suggesting a marked difference in the host response to the virus challenge between bird species.

In summary, we found substantial heterogeneity in viral infection dynamics and host responses to infection between avian hosts and virus subtypes. This has major ramifications for our understanding of infection within the wild bird reservoir, differing roles of different species as hosts, interspecies transmission between different hosts in the wild bird reservoir, and more clarity into the differences and similarities in host response between ducks and chickens. Our experimental design allowed us to address and compare the role of multiple factors for successful wild-type AIV virus infection in chickens and tufted ducks. We propose that the outcome after exposure to AIV in different species depends on several host (physiology and host immune response) and viral factors (route of infection, viral phylogeny, and genetic makeup). These factors need to be integrated to appreciate a holistic understanding of AIV interspecies transmission between birds.

## MATERIALS AND METHODS

### Ethical statement.

All procedures of virus screening and propagation were handled in biosecurity level 2 (BSL2) facilities at the Zoonosis Science Center, Uppsala University. All animal experiments were carried out in strict accordance with a protocol legally approved by the regional board of the animal ethics committee, Sweden (permission number 5.8.18-07998/2017). All animal experiments were conducted in BSL2 animal facilities at the Swedish National Veterinary Institute (SVA).

### Origin of viruses and virus propagation.

Viruses used in the current study were obtained from the Linnaeus University AIV repository (Table S1) and collected and isolated as described in reference 38. Briefly, viruses were isolated from mallards captured at a long-term study site at Ottenby bird observatory, Sweden (56°12′ N 16°24′ E). Viruses were propagated in the allantoic cavity of 11-day-old specific pathogen-free embryonated chicken eggs and harvested fluid was stored at −70°C until further use. The 50% egg infective dose (EID_50_) of the inoculum was determined by infection in specific pathogen-free embryonated chicken eggs (54).

### Strain selection.

We selected AIVs comprising eight subtypes of different phylogenetic lineages that have all been detected in the avian reservoir with different frequencies (Table S1). These include those AIVs that are common and overrepresented in mallards at this study site (H4N6 and H6N2) and elsewhere (H3N8), as well as those that are very infrequently detected and isolated (H8N4, H9N2, H10N1, H11N9, and H15N5). For H15, there have only been 16 detections ever defined (https://www.fludb.org/; accessed 6 September 2020), 6 of which are H15N5 and obtained from this study site.

To understand the phylogenetic relationship of the viruses selected for this study, we downloaded all available HA sequences of H3, H4, H6, H8, H9, H10, H11, and H15 from GenBank until 2010 (accessed 24 August 2021). In addition, available full sequences of internal gene segments of H3, H4, H6, H8, H9, H10, H11, and H15 in the period between 2005 and 2012 were retrieved corresponding to the period from which viruses in this study were collected. The nucleotide sequences were aligned using Geneious Prime work package 2021.1.1 (https://www.geneious.com) and the MAFFT algorithm. Phylogenetic analysis was performed by employing maximum likelihood methodology based on the Akaike criterion after the selection of the best-fit modes using IQ-tree version 2.0 (55). Trees were finally viewed and edited using FigTree v1.4.2 software (http://tree.bio.ed.ac.uk/software/figtree/). In addition, *in silico* comparative analysis of amino acids at or near the HA receptor binding site was performed as described in reference 27.

### Infection dynamics in chickens and tufted ducks.

As a proxy for interspecies transmission events between mallards and chickens or tufted ducks, birds of these two species were challenged with mallard-origin low pathogenic AIV. A total of 72 white leghorn chickens (Gallus gallus *domesticus*) were raised until 3 weeks old at the SVA. Chickens were bought as 1-day-old hatchlings from OVA Production (Sweden), a high-biosecurity company that regularly samples flocks for AIV, Mycoplasma, Salmonella, Egg Drop Syndrome, and Newcastle Disease. The chickens for the experiment were raised completely separated from the other birds at OVA Production until 21 days, wherein they were transferred to SVA. Seventy-two tufted ducks (*Aythya fuligula*; 3 to 6 weeks old) were obtained from Snavelhof, Veeningen, the Netherlands. Birds were divided into groups of four and inoculated with one of the eight viruses or a phosphate-buffered saline (PBS) mock. The oldest tufted ducks were inoculated first to keep the age range at the time of inoculation to 5 to 7 weeks. All animal experiments were carried out in animal rooms in a BSL2 animal facility at the SVA.

To compare the effect of different inoculation routes, 36 individuals of each species were inoculated through either the IE route or combined conjunctival and nasal inoculation (ON), both established routes of infection with AIV in birds (56, 57). For the IE route, we used a 1-mL syringe with a gavage needle, and for the ON route, we gently dripped half the inoculum into the eye and half into the nostril using a 1-mL syringe. Birds were held gently with their beaks closed until the inoculum had entered the bird to ensure they received the inoculum. All inoculums were 10^6^ EID_50_ per 1 mL per bird. For the mock groups, 1 mL of PBS was used per bird.

Birds were monitored daily for clinical signs and mortality until 3 days dpi, when all birds were euthanized by injection of 1 mL of pentobarbital (100 mg). To compare virus shedding patterns via the respiratory and/or digestive tract, oropharyngeal and cloacal swabs were taken every day from all birds and placed in virus transport media (10% glycerol vol/vol; 1× Hanks, 100 U/mL balanced polymyxin sulfate B, 50 U/mL nystatin, 5 g/L lactalbumin, 200 U/mL penicillin, 200 μL/mL streptomycin, 250 μL/mL gentamycin, and MQ water). All birds tested PCR negative in both oropharyngeal and cloacal samples taken the day before inoculation. Tissue samples were collected from the lung, colon, and spleen of euthanized birds at 3 dpi to study virus distribution as well as the transcriptomic host response to infection.

To assess virus transmission from chicken to chicken and from tufted duck to tufted duck, contact transmission experiments were performed using 10^7^ EID_50_ of the H8N4 virus. A total of 21 chickens and 21 tufted ducks from the same source and the same age as described in “Infection dynamics in chickens” below and tufted ducks were used for infection experiments in dedicated rooms for each species. Twelve birds of each species were inoculated oculonasally and nine secondary naive birds of the same species were introduced at 2 dpi and housed with the inoculated birds in the same room. Birds were monitored daily for clinical signs until 9 dpi. Oropharyngeal and cloacal swabs were obtained daily.

### Reverse transcriptase-quantitative real-time PCR.

Viral RNA was extracted from the oropharyngeal and cloacal swab samples using the Maxwell 16 Viral Total Nucleic Acid purification kit on a Maxwell 16 System extraction robot (Promega, Madison, WI, USA). RNA was isolated from the lung, colon, and spleen of all ON inoculated birds using TissueLyser II and RNeasy minikit (Qiagen, Hilden, Germany). Quantification of the viral load (expressed as the EID_50_ equivalent, as defined below) from the swab samples was based on quantification cycle (*C*_q_) values obtained by RT-qPCR targeting the matrix (M) gene (58) and using AgPath-ID one-step RT-PCR reagent kit (Thermo Fisher Scientific, Waltham, MA, USA) on a Bio-Rad CFX1000 real-time PCR system (Bio-Rad, Hercules, CA, USA).

We calculated the EID_50_ equivalent by determining the *C*_q_ values from a known EID_50_ reference virus inoculum. From these data, we generated a standard curve (Fig. S1) correlating these measurements and used this curve to predict EID_50_ equivalents from *C*_q_ data for all virus experiments.

### Whole-genome sequencing and variant detection.

Total viral RNA was extracted from the virus inoculum, all organs with a *C*_q_ value <30, and positive swabs at 3 dpi with a *C*_q_ value <30 using QIAamp viral RNA minikit (Qiagen, Hilden, Germany) according to the kit protocol. Virus whole-genome amplification was undertaken as described by Pohlmann et al. (16). Sample quality control was assessed using a Qubit 4 Fluorometer (using Qubit dsDNA BR assay kit) and Agilent 2200 TapeStation (using Agilent D5000 ScreenTape assay kit). Library construction and sequencing were undertaken at Novogene (Beijing, China). The genomic DNA was randomly fragmented to a size of 350 bp, and then DNA fragments were end polished, A-tailed, ligated with the adapters of Illumina sequencing, and further PCR enriched with primers of P5 and P7 oligonucleotides. The PCR products as the final construct of the libraries were purified followed by a quality control test. Then, 150-bp paired reads were generated with a sequencing depth of 9 to 12 million reads per sample on the Illumina platform NovaSeq 6000.

For variant detection, generated reads were analyzed using Geneious Prime work package (Biomatters, Auckland, New Zealand) through the following pipeline: the primer sequences were trimmed off from the raw reads using the “Trim Ends” Geneious Prime plugin. Next, the trimmed reads were mapped using bowtie2 implemented in Geneious Prime against the whole-genome sequence obtained from the used homologous inoculum, and a consensus sequence was generated for each sample. Finally, variant calling was conducted for the original inoculum and positive detected samples using “variation/SNPs” plugin implemented in Geneious Prime work package on the assembled contigs with a maximum variant *P* value of 10^6^. Variant calling was performed allowing the detection of variant frequencies per nucleotide position down to 5 % with a minimum read count of 50. In addition, separate analyses were conducted for specific sites in the HA and PB2 of putative biological importance (Table S2) allowing for frequencies of 1 % or higher. Nucleotide variants detected at frequencies between 50 and 100% were considered SNPs, and nucleotide variants at frequencies between 1% and 50% were considered iSNV (59, 60). Finally, additional mutational analysis was performed according to https://flusurver.bii.a-star.edu.sg/.

### Serology.

To confirm that all birds were seronegative before infection, blood samples were collected using vacutainer serum plus blood collection tubes (BD, Becton, Dickinson and Company, NJ, USA) from all animals 2 days before the start of the infection experiment. Serum was recovered by centrifugation of the tubes at 1,000 *g* for 5 min. Samples were examined for AIV-specific antibodies using the influenza A nucleoprotein (NP) antibody competition ELISA kit (Idexx, Hoofddorp, The Netherlands) according to the manufacturer’s instructions.

### Transcriptomics.

Gene expression was quantified in the lung, colon, and spleen for all birds at 3 dpi using QuantSeq 3′ mRNA sequencing (61). For this purpose, 25 mg of each tissue was homogenized using Qiagen TissueLyser II, and RNA was extracted using RNeasy minikit and DNase treated using the RNase-Free DNase Set (Qiagen, Hilden, Germany). The quantity and quality of the RNA were assessed using the Qubit (Qubit RNA BR assay kit, Invitrogen, CA, USA) and the Agilent 2100 Bioanalyzer system (Agilent RNA 6000 Nano Kit, CA, USA). The library preparation was conducted using the QuantSeq 3‘mRNA-Seq Library Prep kit (FWD) (Lexogen Inc., Greenland, USA) according to the manufacturer's instructions. Samples were sequenced (75 bp single-end reads) on the Hiseq 4000 platform (Illumina Inc., CA, USA). The raw QuantSeq reads were trimmed using the BBDuk program in the package BBMap (https://sourceforge.net/projects/bbmap/) to remove adapter sequences, poly-A tails, overrepresented sequences, and low-quality bases with the following parameters: k = 13, ktrim = r, useshortkmers = t, mink = 5, qtrim = r, trimq = 10, and minlength = 20. Read quality before and after trimming was checked using FASTQC 0.11.5 (62). The trimmed sequences were aligned to the chicken reference genome (GRCg6a, Ensembl release 99, accession number GCA_000002315.5) and the tufted duck reference genome (bAytFul2.pri, GenBank release 236, accession number GCA_009819795.1) using STAR 2.5.3a (63). For the STAR indexing step, the tufted duck gff annotation file from NCBI was converted into gtf format using the gffread utility in the Cufflinks software 2.2.1 (64). Read counts per gene were calculated in STAR 2.5.3a.

The Bioconductor DESeq2 v1.24.0 (65) package in R v3.6.1 (RCoreTeam, 2016) was used to explore the data and calculate statistical differences of the expression levels of genes between the control birds and the infected birds for each species and tissue (four individuals per group). Cutoff values for significant genes were set to a false discovery rate (FDR) of <0.05 and to genes that were up- or downregulated more than 10%, based on visual inspection of volcanoplots generated in the EnhancedVolcano package (66) in R v3.6.1 (67).

To allow for comparisons of gene expression levels between chicken and tufted duck, a reciprocal best hit (RBH) analysis was conducted. For this purpose, the DNA sequence information for coding sequences (cds) from chicken (GRCg6a, Ensembl release 99) was downloaded. The gffread utility from the STRINGTIE package (68) was then used to generate a FASTA file with the DNA sequences for the coding sequences in the tufted duck. The coding sequences were reciprocally blasted using blastn (69). RBHs were identified using the python script reciprocal_blast_hits.py https://scriptomika.wordpress.com/2014/01/28/extract-best-reciprocal-blast-matches/.

To compare the response between treatment groups, the overlap of differentially expressed genes between groups treated with different virus subtypes within one species and between groups treated with the same virus subtypes between the two species was visualized using the UpSetR package v 1.4.0 (70) in R v3.6.1 (71).

### Transcriptomic response and gene ontology.

The fold changes of differentially expressed genes associated with innate immune response, transcription, glycosylation, and inflammation were visualized in heatmaps. Significantly differentially expressed genes associated with these functions were identified by filtering the DESeq2 output files for gene names identical to the list disclosed in Table S1 or gene names including any of the search terms disclosed in Table S2 and S3 or gene descriptions including “defensin” or “gallinacin” (for β-defensins/gallinacins). To investigate if the expression level of the target genes differed between individual birds within a treatment group and depending on if virus was detected in the tested organ or not, we further generated heatmaps showing the gene expression level for each individual and the outcomes of virus screening in each tissue. For this purpose, we used read counts normalized by library size and transformed using the normTransform() method implemented in DeSeq2 and displayed the virus 10-log EID_50_ equivalents measured for the same tissue sample. Heatmaps were generated using the package pheatmap v 1.0.12 (72) in R v 3.6.1 (71). Gene ontology and pathway analysis were undertaken for differentially expressed genes (adjusted *P* value ≤0.05) for each virus/bird species/organ. Gene set analysis/overrepresentation analysis was conducted using the Webgestalt web interface (http://webgestalt.org/) searching the gene ontology database for “molecular function,” “cellular component,” and “biological process.” Additionally, pathway analysis was conducted by searching KEGG using the Webgestalt web interface. Ontology terms/pathways with an FDR ≤0.05 were visualized in heatmaps.

### Data availability.

We declare that all data supporting the findings of this study are available within the article and its supplementary information files. The nucleotide sequences generated for variant detection and transcriptomic analyses (RNA-seq) reported in this paper have been deposited into public databases, NCBI Sequence Read Archives, under project number PRJNA664709.
