# Supplementary material for: A Comparison of Host Responses to Infection with Wild-Type Avian Influenza Viruses in Chickens and Tufted Ducks
Source: Microbiol Spectr. 2023 Jun 26;11(4):e02586-22. doi: 10.1128/spectrum.02586-22 (PMC10434033; doi:10.1128/spectrum.02586-22)
Supplement: Supplemental file 1 — Supplemental material. Download spectrum.02586-22-s0001.docx, DOCX file, 11.0 MB [file spectrum.02586-22-s0001.docx]

**Supplementary Information for**

**A comparison of host responses to infection with wild-type avian influenza viruses in chickens and tufted ducks**

Mahmoud M. Naguib^1^, Per Eriksson^1^, Elinor Jax ^2,3^, Michelle Wille^1#^, Cecilia Lindskog^4^, Caroline Bröjer^5^, Jonas Waldenström^6^, Robert H. S. Kraus ^2,3^, Göran Larson^7,8^ , Åke Lundkvist^1^, Björn Olsen^9^, Josef D. Järhult^9*^, Patrik Ellström^9*^

Corresponding Author

Patrik Ellstrom: [patrik.ellstrom@medsci.uu.se](mailto:patrik.ellstrom@medsci.uu.se)

**This PDF file includes:**

Figures S1 to S8

Tables S1 to S6

**Supplementary figures**

**
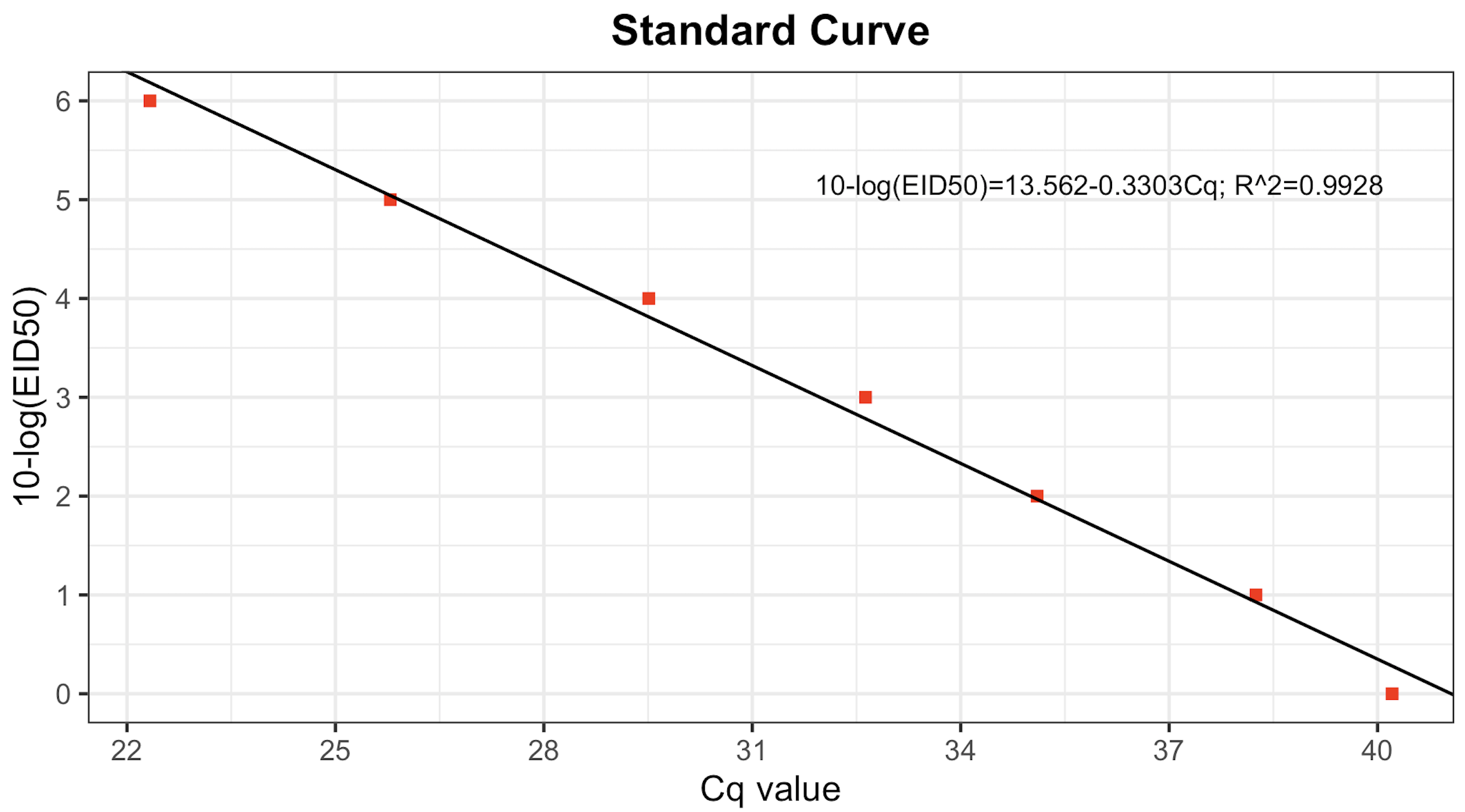
**

**Figure S1. Rt-qPCR standard curve for the titration of the inoculum**. Standard curve was obtained from serial 10-fold dilutions of the inoculum. Red squares showing the Cq values.  Correlation coefficient R^2^ (0.998)

**Figure S2. Low pathogenic avian influenza phylogeny of PB2, PB1, PA, NP, M, and NS genes.** Sequences obtained from poultry including chickens, quails, turkeys, and pheaseants are coloured in red; but do not include domestic ducks due to metadata limitations. Sequences obtained from *Anas* ducks are coloured in blue. Sequences obtained fom Charadriiformes (shorebirds, gulls, alcidae) are colored in green. Sequences obtained from Anseriformes minus *Anas* ducks, and all instances wherein metadata was missing (eg “duck”) are colored in grey. Scale bar indicates number of nucleotide substitutions per site.

**
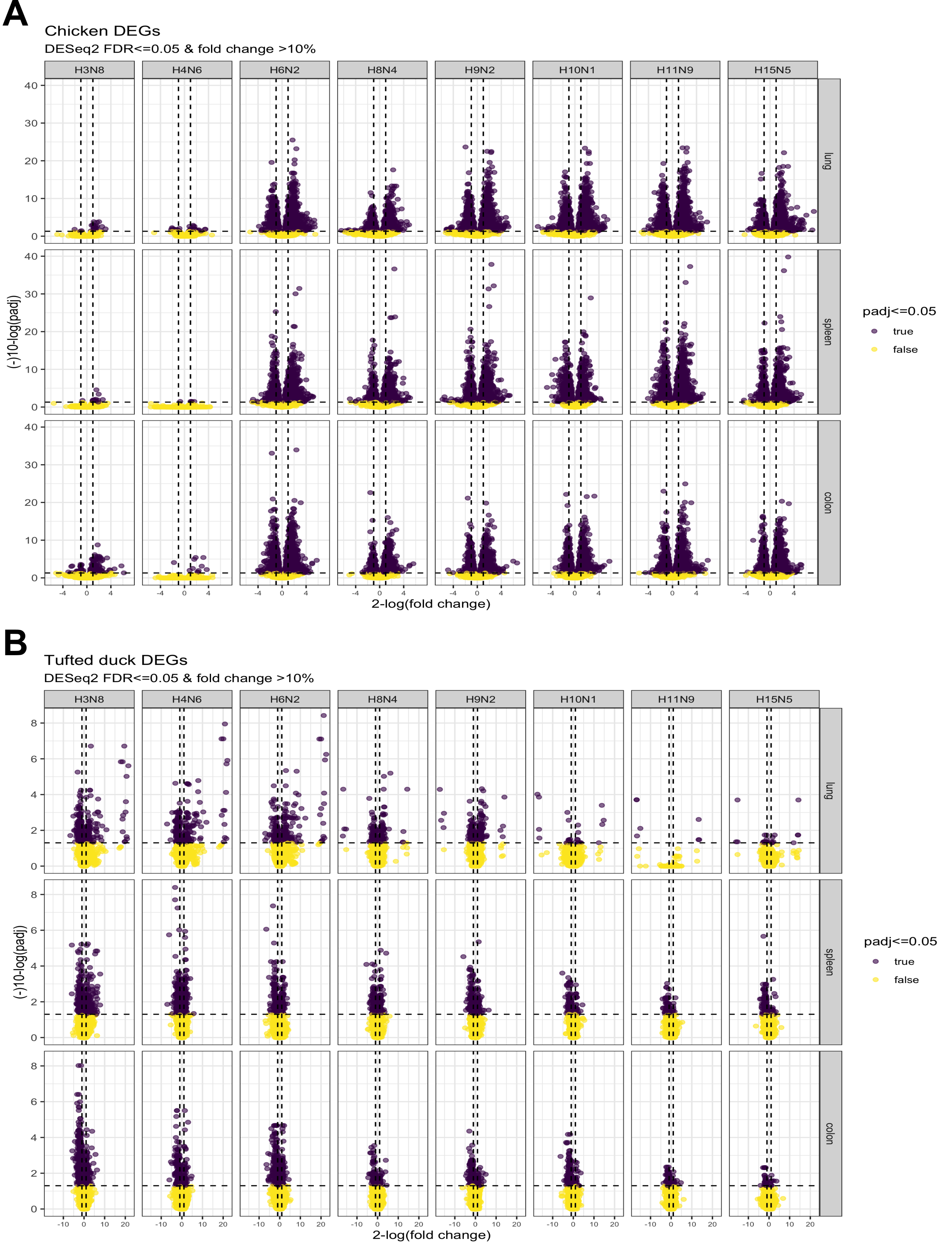
Figure S3. Volcano plots obtained from chickens (a) and tufted ducks (b) infection experiments.** Volcano plot showing DEGs for H3N8, H4N6, H6N2, H8N4, H9N2, H10N1, H11N9, and H15N5. The x-axis represents the log2 values of the fold change observed for each mRNA transcript, and the y-axis represents the log10 values of the adjusted p-values <=0.05.


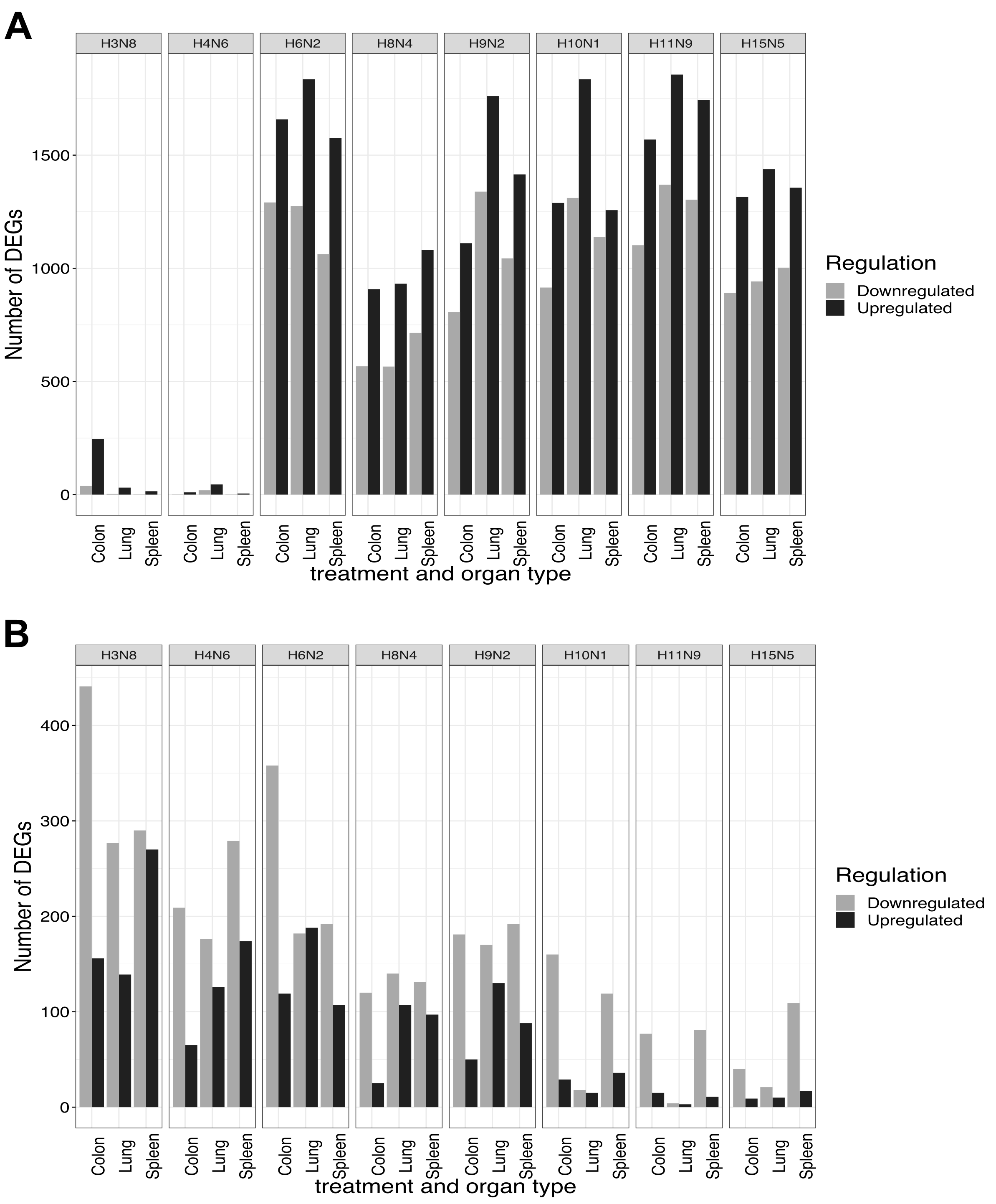


**Figure S4.** **Differentially expressed genes in infected chickens (a) and tufted ducks (b).** Number of differentially expressed genes (DEGs) in each tissue type for each virus subtype in chicken (a) and tufted ducks (b). Genes with an adjusted p-value (FDR) of ≤ 0.05 that were up- or down regulated at least 10% were considered differentially expressed.


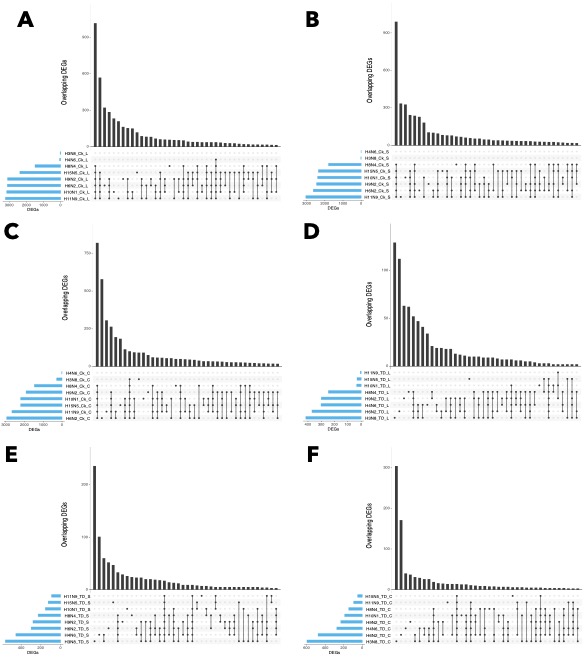


**Figure S5.** **UpSet plot of the intersection of differentially expressed genes in chickens (a-c) and tufted ducks (d-e) tissues displayed for each virus subtype.** Ck = chickens*,* TD= tufted ducks*,* L = lung, S = spleen C = colon*.* The nature of a given intersection is indicated by the dots below the bar plot. For instance, the genes in the 9^th^ column in A are differentially expressed in chicken lung following treatment with H10N1 and H11N9, whereas the genes in the 3^rd^ column in A were differentially expressed in chicken lung in response to H6N2 only.

**
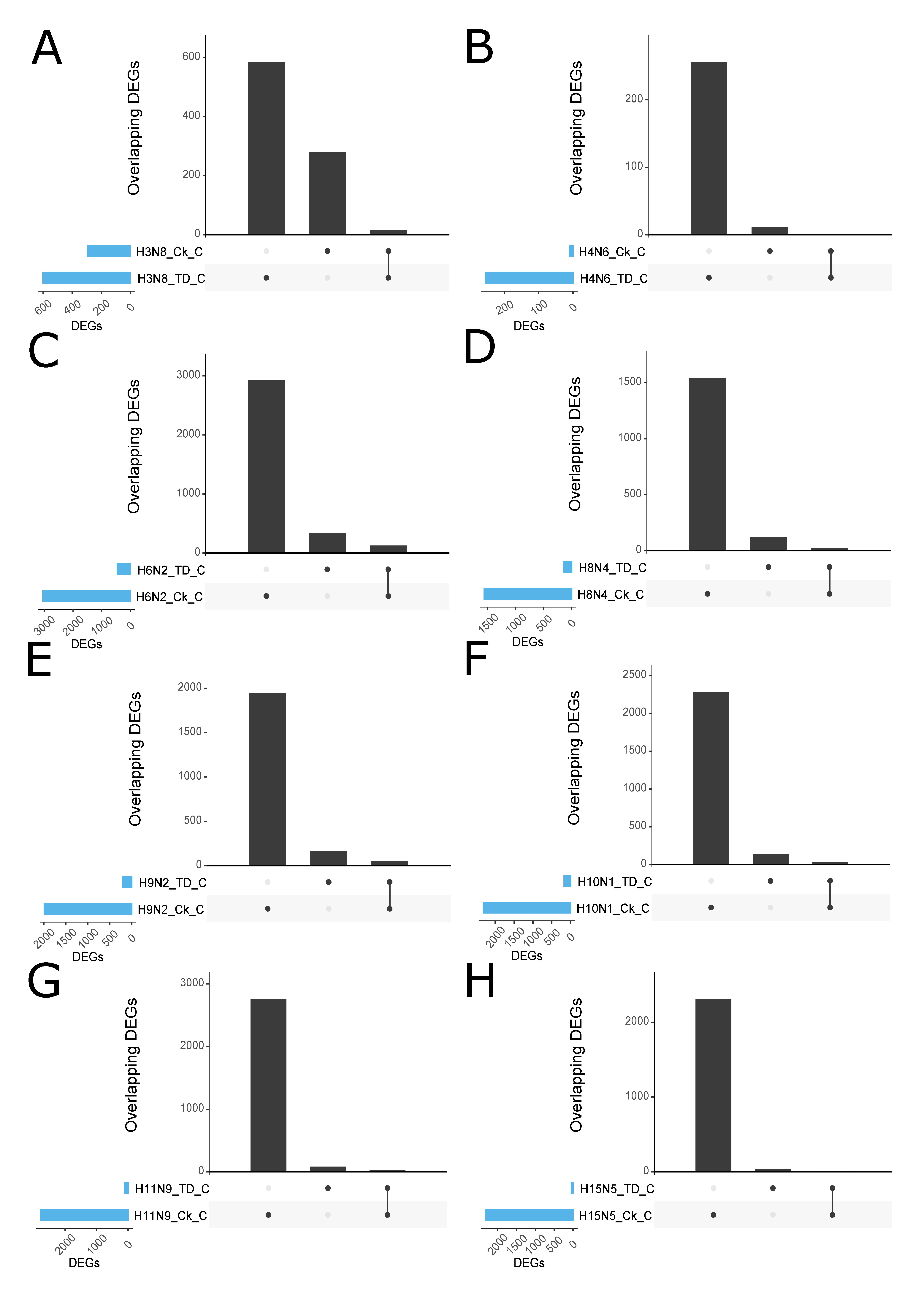
**

**Figure S6. UpSet plot of the intersection of differentially expressed orthologous genes in tufted duck and chicken colon tissue, dispalyed for each virus subtype**. A) H3N8, B) H4N6, C) H6N2, D) H8N4, E) H9N2, F) H10N1, G) H11N9, and H) H15N5. Ck = chickens*,* TD= tufted ducks*.* The nature of a given intersection is indicated by the dots below the bar plot. For instance, the genes in the first column in A are differentially expressed in tufted duck (TD) but not in chicken (Ck). Orthologs represent reciprocal best blast hits between the two species.

**
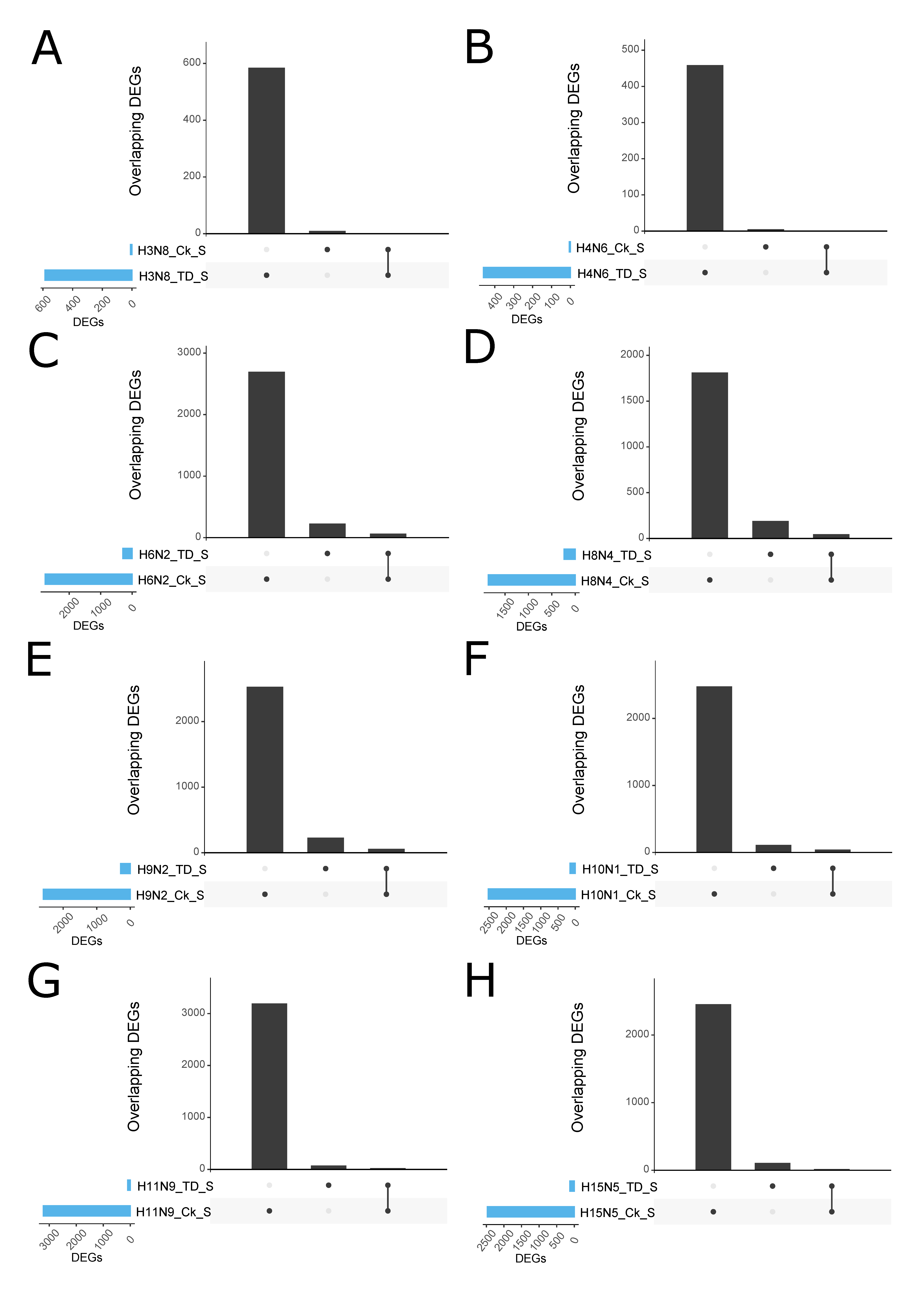
**

**Figure S7. UpSet plot of the intersection of differentially expressed orthologous genes in tufted duck and chicken spleen tissue, displayed for each virus subtype**. A) H3N8, B) H4N6, C) H6N2, D) H8N4, E) H9N2, F) H10N1, G) H11N9, and H) H15N5. Ck = chickens*,* TD= tufted ducks*.* The nature of a given intersection is indicated by the dots below the bar plot. For instance, the genes in the first column in A are differentially expressed in tufted duck (TD) but not in chicken (Ck). Orthologs represent reciprocal best blast hits between the two species.

**
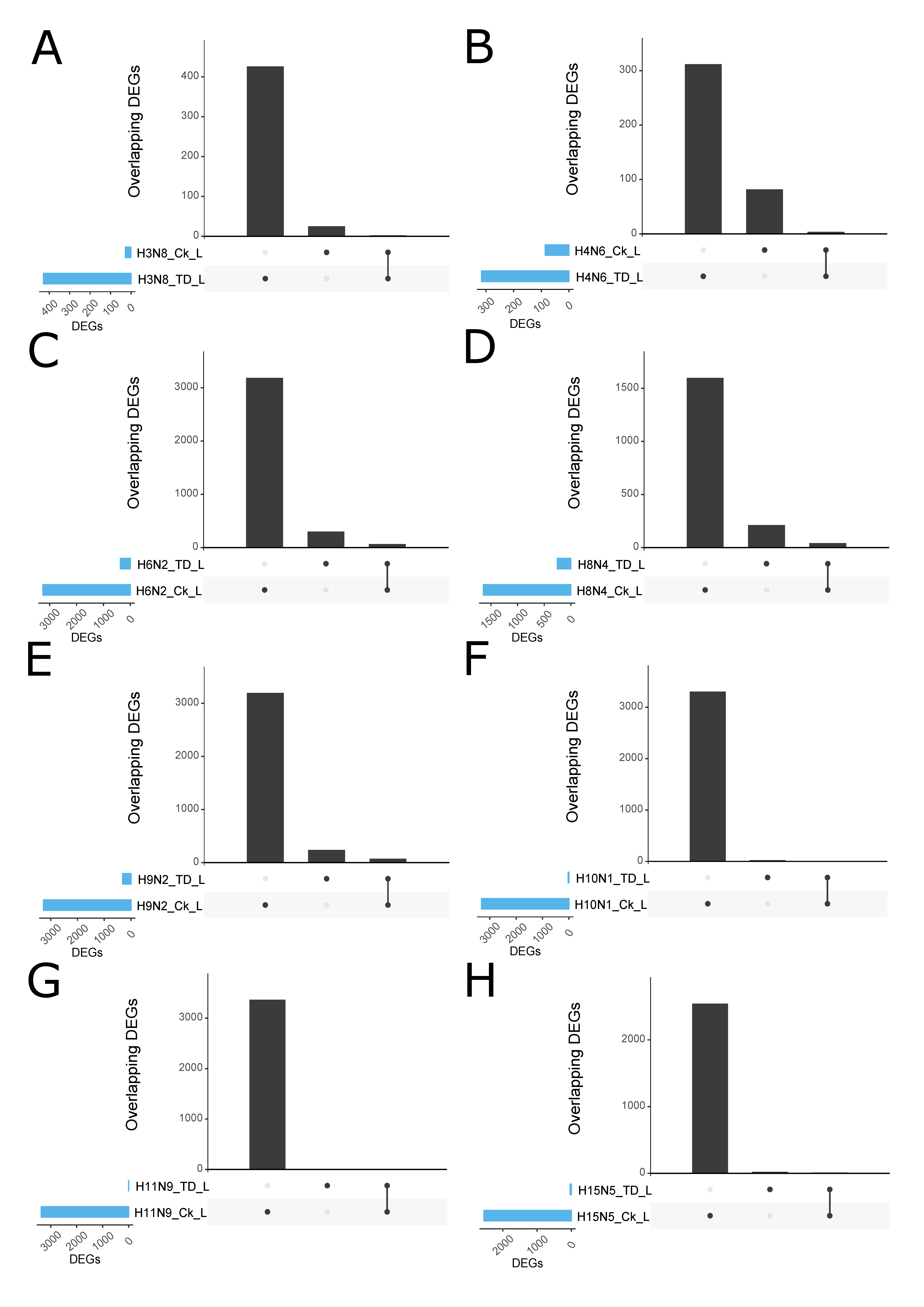
**

**Figure S8. UpSet plot of the intersection of differentially expressed orthologous genes in tufted duck and chicken lung tissue, displayed for each virus subtype**. A) H3N8, B) H4N6, C) H6N2, D) H8N4, E) H9N2, F) H10N1, G) H11N9, and H) H15N5. Ck = chickens*,* TD= tufted ducks*.* The nature of a given intersection is indicated by the dots below the bar plot. For instance, the genes in the first column in A are differentially expressed in tufted duck (TD) but not in chicken (Ck). Orthologs represent reciprocal best blast hits between the two species.

**Figure S9**. Heatmap showing gene expression levels for individual **chickens** for selected immune genes in **lung, spleen and colon** tissue. The sequencing read counts are normalized by library size and transformed (log2 + 1). The top panels show which treatment group each bird belong to and whether AIV RNA was detected in the tissue type for which the gene expression data was generated in for each bird.

**Figure S10**. Heatmap showing gene expression levels for individual **chickens** for selected glycosylation and transcription genes in **lung, spleen and colon**. The sequencing read counts are normalized by library size and transformed (log2+1). The top panels show which treatment group each bird belong to and whether AIV RNA was detected in the tissue type for which the gene expression data was generated in for each bird.

**Figure S11**. Heatmap showing gene expression levels for individual **tufted ducks** for selected glycosylation and transcription genes in **lung, spleen and colon**. The sequencing read counts are normalized by library size and transformed (log2+1). The top panels show which treatment group each bird belong to and whether AIV RNA was detected in the tissue type for which the gene expression data was generated in for each bird.

**Supplementary tables**

**Table S1.** Investigated virus isolates.

| Subtype | Virus Designation | Accession number^#^ |
| --- | --- | --- |
| H3N8 | A/Mallard/Sweden/101487/2009 | EPI845220-22 |
| H4N6 | A/Mallard/Sweden/80148/2008 | CY165570-77* |
| H6N2 | A/Mallard/Sweden/99825/2009 | EPI462903, EPI462995  EPI463087, EPI463131  EPI463271, EPI463315  EPI463406, EPI463547 |
| H8N4 | A/Mallard/Sweden/58256/2006 | EPI619166, EPI619165  EPI613872, EPI619160  EPI619163, EPI619162  EPI619161, EPI619164 |
| H9N2 | A/Mallard/Sweden/67860/2007 | CY184149-56* |
| H10N1 | A/Mallard/Sweden/102087/2009 | CY183839-46* |
| H11N9 | A/Mallard/Sweden/102103/2009 | CY184189-96 |
| H15N5 | A/Mallard/Sweden/139647/2012 | MF148060, MF147769 MF147831, MF147992 MF147041, MF148018 MF147434, MF146217* |

**^#^** The nucleotide sequences generated for variant detection including the inocula reported in the table have been deposited into public databases, NCBI Sequence Read Archives, under project number PRJNA664709.

*GenBank accession number

**Table S2.** Comparative analyses among different biologically important sites for viruses used in this study (4)

| **Strain name ^a^** | **HA (Receptor binding sites) (H3 numbering)** | | | | | | | | | | | | | | | | **HA (Cleavage sites)** | **PB2 (Enhance virus replication or virulence to mammals)** | | | | | **PA (Affect BXA susceptibility)** | | **M2** |
| --- | --- | --- | --- | --- | --- | --- | --- | --- | --- | --- | --- | --- | --- | --- | --- | --- | --- | --- | --- | --- | --- | --- | --- | --- | --- |
|  | **135** | **138** | **155** | **158** | **159** | **160** | **173** | **183** | **186** | **189** | **190** | **221** | **222** | **226** | **227** | **228** |  | **526** | **588** | **591** | **627** | **701** | **28** | **63** | **31** |
| A/Mallard/Sweden/101487/2009_H3N8 | G | A | T | G | N | A | N | H | S | Q | E | P | W | Q | S | G | PEKQTRGLF | K | A | Q | E | D | P | V | S |
| A/Mallard/Sweden/80148/2008_H4N6 | K | A | V | G | N | A | D | H | S | T | E | P | W | Q | S | G | PEKASRGLF | K | T | Q | E | D | P | V | S |
| A/Mallard/Sweden/99825/2009_H6N2 | V | A | I | S | A | A | N | H | P | N | E | P | A | Q | R | G | PQIETRGLF | K | A | Q | E | D | P | V | S |
| A/Mallard/Sweden/58256/2006_H8N4 | T | A | T | P | D | T | D | H | P | K | E | P | L | Q | Q | G | PSIEPKGLF | K | A | Q | E | D | P | V | S |
| A/Mallard/Sweden/67860/2007_H9N2 | T | A | T | S | N | S | G | H | P | T | E | P | L | Q | Q | G | PAASDRGLF | K | A | Q | E | D | P | V | S |
| A/Mallard/Sweden/102087/2009_H10N1 | T | A | V | G | Q | N | T | H | S | Q | E | P | Q | Q | S | G | PELMQRGLF | K | T | Q | E | D | P | V | S |
| A/Mallard/Sweden/102103/2009_H11N9 | V | A | I | S | G | T | G | H | A | K | E | P | K | Q | A | G | PAIASRGLF | K | A | Q | E | D | P | V | S |
| A/Mallard/Sweden/139647/2012_H15N5 | V | A | L | N | Q | V | K | H | S | E | E | P | K | Q | A | G | PEKTHTRGLF | K | A | Q | E | D | P | V | S |

**Table S3.** Variant detection after influenza A virus infection using virus inoculum as a reference.

| **Virus** | **Sample ID** | **Gene segment** | **Position** | | **Change** | **Polymorphism** | **Variant frequency** | **Reference frequency** | **Variant in inoculum** | **AA** | **Function^1^** |
| --- | --- | --- | --- | --- | --- | --- | --- | --- | --- | --- | --- |
| H3 | Ck/H3/2OP 3d.p.i. | PB2 | 47 | | C -> A | SNP | 85.6% | 14.2% | 0% | T16N | binding other viral protein(s) |
|  |  | HA^2^ | 248 | | T -> C | iSNV | 10.7% | 88.9% | 0% | I67T | binding small ligand(s) |
|  |  |  | 444 | | G -> A | iSNV | 2.2% | 97.7% | 0% | Silent | - |
|  |  |  | 445 | | A -> G | iSNV | 1.4% | 98.5% | 1.1% | N133D | increase in binding to α2,6-SAs |
|  |  |  | 1236 | | A -> G | iSNV | 6.6% | 93.3% | 0.1% | Silent | - |
|  |  | NP | 157 | | G -> A | iSNV | 5.3% | 94.6% | 0% | E53K | binding small ligand(s) |
|  |  |  | 785 | | C -> T | iSNV | 12.5% | 87.5% | 0% | S262F | No report |
|  |  | NA | 612 | | T -> C | SNP | 99.9% | 0.01% | 0.3% | Silent | - |
|  | Ck/H3/4OP 3d.p.i. | PA | 960 | | C -> A | SNP | 99.1% | 0.6% |  | Silent | - |
|  |  | HA | 406 | | T -> C | iSNV | 3.8% | 96.0% | 0% | F120L | viral oligomerization interfaces; binding small ligand(s); involved in a T-cell epitope presented by MHC molecules; antibody recognition sites |
|  |  |  | 547 | | A -> G | iSNV | 1.3% | 98.6% | 0% | T167A | antibody recognition sites; binding small ligand(s); viral oligomerization interfaces |
|  |  |  | 983 | | A -> G | iSNV | 5.6% | 94.3% | 0.2% | N312S | involved in binding host protein(s); binding small ligand(s); viral oligomerization interfaces; involved in a T-cell epitope presented by MHC molecules |
|  |  | NP | 757 | | A -> G | iSNV | 22.4% | 77.4% | <0.1% | I253V | No report |
|  |  | M | 41 | | T -> C | iSNV | 10.4% | 89.5% | <0.1% | I14T | viral oligomerization interfaces |
| H6 | Ck/H6/colon | PB1 | 271 | | G -> A | iSNV | 11.9% | 87.9% | 0% | A91T, silent in PB1-F2 | No report |
|  |  | PA | 187 | | G -> T | SNP | 99.7% | 0.03% | 5.0% | V63L | Associated with increase of virulence |
|  |  | HA | 513 | | A -> T | iSNV | 1.8% | 98.0% | 0% | Silent | - |
|  |  |  | 594 | | T -> C | iSNV | 1.2% | 98.8% | 0.3% | Silent | - |
|  |  |  | 1458 | | T -> C | iSNV | 6.5% | 93.4% | 0.1% | Silent | - |
|  |  |  | 1530 | | T -> C | iSNV | 6.2% | 93.7% | 0.1% | Silent | - |
|  |  |  | 1550 | | T -> C | iSNV | 6.0% | 93.9% | 0.1% | I502T | viral oligomerization interfaces |
|  | Ck/H6/spleen | PB2 | 225 | | G -> A | iSNV | 42.2% | 57.8% | 0.4% | Silent | - |
|  |  |  | 1387 | | A -> G | iSNV | 5.3% | 94.0% | 0.1% | I463V | No report |
|  |  | PB1 | 81 | | T -> C | iSNV | 32.6% | 67.4% | 0.2% | Silent | - |
|  |  |  | 84 | | C -> T | iSNV | 97.3% | 2.7% | 0.1% | Silent | - |
|  |  | PA | 120 | | G -> A | iSNV | 10.9% | 88.8% | 0.1% | Silent | - |
|  |  |  | 187 | | G -> T | SNP | 93.0% | 7.0% | 5.0% | V63L | Associated with increase of virulence |
|  |  |  | 207 | | T -> C | iSNV | 5.8% | 94.2% | 0.2% | Silent | - |
|  |  |  | 526 | | T -> C | iSNV | 10.5% | 89.2% | 0.3% | F176L | viral oligomerization interfaces |
|  |  |  | 1983 | | G -> A | iSNV | 10.2% | 89.8% | 0.2% | Silent | - |
|  |  | HA | 537 | | A -> G | iSNV | 1.0% | 98.9% | 0.1% | Silent | - |
|  |  |  | 577 | | T -> C | iSNV | 1.1% | 98.9% | 0.1% | W180R | No report |
|  |  |  | 665 | | T -> C | iSNV | 1.8% | 98.1% | 0.1% | M209T | viral oligomerization interfaces; antibody recognition sites |
|  |  | NP | 650 | | T -> C | iSNV | 12.2% | 87.5% | 0.1% | I217T | viral oligomerization interfaces |
|  |  | M | 726 | | G -> A | SNP | 83.1% | 16.9% | 0% | M2: S13N | No report |
|  |  |  | 815 | | C -> T | SNP | 84.9% | 14.8% | 0% | M2: L43F | drug binding; binding small ligand(s); viral oligomerization interfaces |
|  |  |  | 910 | | A -> G | SNP | 79.8% | 20.0% | 0.2% | Silent | - |
|  |  |  | 913 | | G -> A | SNP | 79.8% | 20.0% | 0% | Silent | - |
|  |  | NS | 314 | | T -> A | iSNV | 17.2% | 82.5% | 0% | NS1: L105H | binding host protein(s); viral oligomerization interfaces |
|  | Ck/H6/lung | PB1 | 270 | | A -> G | iSNV | 6.2% | 93.7% | 0.4% | PB1-F2: K59R | No report |
|  |  |  | 1986 | | T -> C | iSNV | 16.6% | 83.3% | 0% | Silent | - |
|  |  |  | 1993 | | T -> C | iSNV | 16.7% | 83.2% | 0.4% | S665P | binding other viral protein(s) |
|  |  |  | 2016 | | T -> C | iSNV | 17.2% | 82.6% | 0.8% | Silent | - |
|  |  |  | 2161 | | A -> C | iSNV | 8.7% | 91.1% | 8.6% | Silent | - |
|  |  | PA | 187 | | G -> T | SNP | 99.7% | - | 5.0% | V63L | Associated with increase of virulence |
|  |  | M | 726 | | G -> A | SNP | 99.5% | 0.4% | 0% | M2: S13N | No report |
|  |  |  | 815 | | C -> T | SNP | 99.5% | 0.3% | 0% | M2: L43F | drug binding; binding small ligand(s); viral oligomerization interfaces |
|  |  |  | 910 | | A -> G | SNP | 99.7% | 0.03% | 0.2% | Silent | - |
|  |  |  | 913 | | G -> A | SNP | 99.7% | 0.1% | 0% | Silent | - |
|  | Ck/ H6/3OP 3d.p.i. | PB2 | 42 | | T -> C | iSNV | 6.0% | 94.0% | 0.3% | Silent | - |
|  |  | PB1 | 2161 | | A -> C | iSNV | 12.1% | 87.7% | 8.6% | Silent | - |
|  |  | PA | 1404 | | C -> T | SNP | 99.8% | 0.02% | 0% | Silent | - |
|  |  | HA | 1300 | | G -> A | SNP | 99.7% | 0.2% | 15.8% | D419N | viral oligomerization interfaces; binding small ligand(s) |
|  |  |  | 1339 | | G -> A | iSNV | 27.1% | 71.8% | 0% | E432K | viral oligomerization interfaces; binding host protein(s); binding small ligand(s) |
|  |  | M | 731 | | T -> G | iSNV | 28.2% | 71.7% | 0.4% | M1: M244R  M2: W15G | No report  No report |
|  |  | NS | 82 | | G -> A | iSNV | 6.2% | 93.7% | 0% | NS1: G28S | viral oligomerization interfaces; binding small ligand(s) |
|  |  |  | 123 | | G -> A | iSNV | 6.3% | 93.6% | 0% | Silent | - |
|  |  |  | 154 | | C -> A | iSNV | 7.4% | 92.5% | 0% | L52M | viral oligomerization interfaces; binding small ligand(s) |
|  |  |  | 263 | | G -> A | iSNV | 6.0% | 93.9% | 0.1% | R88H | viral oligomerization interfaces; binding small ligand(s); binding host protein(s) |
|  |  |  | 611 | | G -> A | iSNV | 7.0% | 92.9% | 0% | NS1: R204K  NS2: E47K | viral oligomerization interfaces  No report |
|  | TD/H6/colon | PB2 | 1,753 | | C -> G | SNP | 85.7% | 14.3% | 0% | P 585 A | viral oligomerization interfaces |
|  |  | PB1 | 84 | | C -> T | SNP | 92.9% | 7.1% | 0.1% | Silent | - |
|  |  | M | 726 | | G -> A | SNP | 92.4% | 7.6% | 0% | M2: S13N | No report |
|  |  |  | 767 | | G -> A | SNP | 51.8% | 48.2% | 0.1% | M2: V27I | drug binding (resistance); viral oligomerization interfaces; binding small ligand(s) |
|  |  |  | 815 | | C -> T | iSNV | 34.9% | 64.3% | 0% | M2: L43F | drug binding; binding small ligand(s); viral oligomerization interfaces |
|  |  |  | 910 | | A -> G | iSNV | 36.6% | 63.4% | 0.2% | Silent | - |
|  |  |  | 913 | | G -> A | SNP | 84.0% | 16.0% | 0% | Silent | - |
|  | TD/H6/ Spleen | PB2 | 683 | | A -> G | SNP | 99.9% | 0.03% | 0.1% | Y228C | No report |
|  |  | HA | 458 | | A -> G | iSNV | 1.3% | 98.7% | 0% | Y141C | antibody recognition sites; viral oligomerization interfaces; binding small ligand(s) |
|  |  |  | 590 | | A -> G | iSNV | 1.8% | 98.1% | 0.1% | H184R | antibody recognition sites ; viral oligomerization interfaces; binding small ligand(s) |
|  |  | NP | 198 | | G -> A | SNP | 99.8% | 0.1% | 0% | M66I | No report |
|  |  |  | 495 | | T -> C | SNP | 99.9% | 0.02% | 0.2% | Silent | - |
|  |  |  | 957 | | T -> A | SNP | 99.7% | 0.04% | 0% | N319K | related to virulence, host specificity shift |
|  |  | M | 726 | | G -> A | SNP | 99.7% | 0.3% | 0% | M2: S13N | No report |
|  |  |  | 763 | | T -> C | SNP | 99.8% | 0.03% | 0.2% | Silent | - |
|  |  |  | 815 | | C -> T | SNP | 99.6% | 0.1% | 0% | M2: L43F | - drug binding - binding small ligand(s) - viral oligomerization interfaces |
|  |  |  | 910 | | A -> G | SNP | 99.8% | 0.02% | 0.2% | Silent | - |
|  |  |  | 913 | | G -> A | SNP | 98.3% | 1.6% | 0% | Silent | - |
|  | TD/H6/ OP 3d.p.i. | PA | 187 | | G -> T | iSNV | 38.7% | 61.2% | 5.0% | V63L | Associated with increase of virulence |
|  |  | HA | 374 | | A -> G | SNP | 52.1% | 47.8% | 29.0% | E116G | viral oligomerization interfaces; binding small ligand(s); involved in a T-cell epitope presented by MHC molecules |
|  |  |  | 547 | | A -> C | SNP | 57.5% | 42.3% | 3.7% | N170H | viral oligomerization interfaces; binding small ligand(s) |
|  | TD/H6/CL 3d.p.i. | PA | 187 | | G -> T | SNP | 99.8% | 0.02% | 5.0% | V 63 L | Virulence |
|  |  | HA | 547 | | A -> C | SNP | 99.9% | 0.01% | 3.7% | N170H | viral oligomerization interfaces; binding small ligand(s) |
| H8 | TD/H8/Spleen |  | | None | | | | | | | - |
|  | TD/H8/3OP 3d.p.i. | PB2 | 321 | | T -> G | iSNV | 16.6 | 83.1 | 0.1% | S107R | No report |
|  |  |  | 1402 | | G -> A | SNP | 67.2% | 32.7% | 15.2% | A468T | viral oligomerization interfaces |
|  |  |  | 1932 | | G -> A | SNP | 67.0% | 32.9% | 7.3% | Silent | - |
|  |  |  | 2153 | | A -> G | SNP | 66.6% | 33.3% | 25.7% | K718R | binding host protein(s) |
|  |  | PB1 | 1170 | | A -> G | iSNV | 21.6 | 78.3 | 25.8% | Silent | - |
|  |  |  | 1548 | | G -> A | iSNV | 21.5 | 78.3 | 25.9% | Silent | - |
|  |  |  | 1915 | | T -> C | SNP | 68.5% | 31.4% | 0.4% | S639P | No report |
|  |  | PA | 772 | | G -> A | iSNV | 14.0% | 85.9% | 0.1% | E258K | No report |
|  |  |  | 2030 | | A -> G | SNP | 60.0% | 39.9% | 0.2% | E677G | No report |
|  |  | HA | 366 | | C -> A | SNP | 80.1% | 19.8% | 14.8% | F112L | No report |
|  |  |  | 997 | | A -> G | SNP | 76.3% | 23.3% | 14.3% | I318V | antibody recognition sites; binding host protein(s); binding small ligand(s); viral oligomerization interfaces involved in a T-cell epitope presented by MHC molecules |
|  |  |  | 1269 | | G -> A | iSNV | 11% | 88.7% | 2.0% | M408I | viral oligomerization interfaces; binding small ligand(s) |
|  |  |  | 1270 | | A -> G | iSNV | 11.1% | 88.7% | 0.3% | I409V | viral oligomerization interfaces |
|  |  | NA | 354 | | G -> A | iSNV | 5.1% | 94.8% | 0.1% | Silent | - |
| H9 | TD/H9/4OP 3d.p.i. | PB1 | 2161 | | A -> C | iSNV | 12.0% | 87.5% | 9.0% | Silent | - |
|  |  | PA | 160 | | A -> G | iSNV | 16.0% | 83.9% | 0.1% | I54V | viral oligomerization interfaces |
|  |  | HA | 1001 | | C -> T | iSNV | 5.4% | 94.5% | 0.1% | A325V | antibody recognition sites; Viral oligomerization interfaces; binding small ligand(s) |
|  |  |  | 1108 | | G -> A | iSNV | 11.6% | 88.2% | 0% | V361I | antibody recognition sites; Viral oligomerization interfaces |
|  |  | M | 953 | | G -> A | iSNV | 26.5% | 73.3% | 0.1% | M2: G89S | related to host specificity shift |
| H10 | Ck/H10/2OP 3d.p.i. | HA | 493 | | A -> G | iSNV | 1.0% | 98.9% | 0% | K156E | related to virulence, antigenic drift / escape mutant.  Involved in antibody recognition sites |
|  |  | NP | 1326 | | T -> C | iSNV | 5.0% | 95.0% | 0.1% | Silent | - |
|  |  | NA | 1335 | | T -> C | iSNV | 6.2% | 93.5% | 0% | Silent | - |
|  |  | M | 123 | | T -> C | iSNV | 5.4% | 94.6% | 0.2% | Silent | - |
|  |  |  | 863 | | T -> C | iSNV | 5.9% | 93.9% | 0.2% | Silent | - |
|  |  |  | 931 | | G -> A | iSNV | 5.5.% | 94.4% | 0.1% | Silent | - |
|  |  | NS | 21 | | A -> G | iSNV | 7.0% | 92.9% | 0% | Silent | - |
|  | TD/H10/3OP 3d.p.i. | PB2 | 2133 | | C -> T | iSNV | 5.7% | 94.1% | 0.1% | Silent | - |
|  |  | PB1 | 2161 | | A -> C | iSNV | 8.6% | 91.2% | 6.8% | Silent | - |
|  |  | HA | 406 | | T -> C | iSNV | 2.5% | 97.5% | 0% | S128P | antibody recognition sites; host specificity shift |
|  |  |  | 665 | | A -> G | iSNV | 1.5% | 98.4% | 0.6% | N212S | antibody recognition sites; viral oligomerization interfaces |
|  |  | NA | 1307 | | T -> C | iSNV | 5.6% | 94.2% | 0.5% | I436T | related to mild drug resistance and creates a new potential N-glycosylation site |
|  |  | M | 841 | | T -> C | iSNV | 13.5% | 86.3% | 0.1% | Silent | - |
|  |  | NS | 272 | | C -> T | iSNV | 14.4% | 85.5% | 0% | NS1: T91I | binding host protein(s); binding small ligand(s); in a T-cell epitope presented by MHC molecules |

^1^Mutational analysis was performed according <https://flusurver.bii.a-star.edu.sg>

^2^HA numbering is according to the H3 numbering system

^3^ “–“ is equal to 0%

**Table S4. Genes used for construction of heat map showing significant DEGs related to innate immunity (Figure 2)**

| Gene  Name^a^ | Lung | | | | | | | | Spleen | | | | | | | | Colon | | | | | | | |
| --- | --- | --- | --- | --- | --- | --- | --- | --- | --- | --- | --- | --- | --- | --- | --- | --- | --- | --- | --- | --- | --- | --- | --- | --- |
|  | H3 | H4 | H6 | H8 | H9 | H10 | H11 | H15 | H3 | H4 | H6 | H8 | H9 | H10 | H11 | H15 | H3 | H4 | H6 | H8 | H9 | H10 | H11 | H15 |
| AvBD1 | - | - | - | - | - | - | - | - | - | - | -1,34 | - | - | - | -1,72 | - | - | - | - | - | - | - | - | -3,74 |
| AvBD4 | - | - | - | - | - | - | -3,41 | - | - | - | - | - | - | - | - | - | - | - | - | - | - | - | - | - |
| AvBD6 | - | - | - | - | - | - | - | - | - | - | - | - | - | - | -2,34 | - | - | - | - | - | - | - | - | - |
| DEFB4A | - | - | -2,18 | - | - | - | - | - | - | - | -2,05 | - | -1,46 | -1,77 | -1,96 | -1,35 | - | - | - | - | - | - | - | - |
| IFIT5 | - | - | 2,11 | - | - | - | - | - | - | - | - | - | - | - | - | - | - | - | - | - | - | - | - | - |
| IL8 | - | - | - | - | - | - | - | - | - | - | - | - | - | -2,95 | - | - | - | 3,70 | - | - | 2,34 | - | - | - |
| IRF7 | - | - | - | -1,16 | -1,10 | -1,05 | - | - | - | - | - | - | - | - | - | - | - | - | - | - | - | - | - | - |
| LSM14A | - | - | - | - | - | - | - | - | - | - | -0,48 | -0,64 | -0,53 | - | - | -0,59 | - | - | -0,56 | -0,53 | - | -0,48 | -0,52 | -0,54 |
| LSM14B | - | - | - | - | - | - | - | - | - | - | - | - | - | - | - | - | - | - | - | - | - | - | - | 2,50 |
| MAVS | - | - | -1,28 | - | -1,11 | -0,92 | -1,25 | -0,85 | - | - | -1,03 | -0,79 | -0,85 | - | -0,91 | -1,13 | - | - | -0,76 | -1,05 | -1,06 | -1,08 | -1,10 | -0,88 |
| OASL | - | - | 2,33 | - | - | - | - | - | - | - | 3,37 | - | - | - | - | - | - | - | 1,80 | - | - | - | - | - |
| RSAD2 | - | - | 2,18 | - | - | - | - | - | - | - | 2,22 | - | - | - | - | - | - | - | - | - | - | - | - | - |
| TICAM1 | - | - | -2,04 | - | -2,01 | -1,98 | -2,08 | -1,69 | - | - | -1,42 | - | -1,16 | -2,60 | -1,72 | - | - | - | -2,12 | - | - | -0,97 | -1,22 | - |
| TLR3 | - | - | 1,21 | - | - | - | - | - | - | - | - | - | - | - | 1,52 | - | - | - | 1,88 | 1,45 | 1,61 | 1,50 | 1,46 | 1,43 |
| TRIM25 | - | - | 1,75 | - | 1,06 | 1,14 | 1,10 | - | - | - | 1,95 | - | 1,07 | 1,53 | 1,56 | 1,41 | 1,01 | - | 1,39 | 1,33 | 0,91 | 1,14 | 1,18 | 1,19 |
| CCL19 | - | - | - | - | - | - | - | - | - | - | - | - | - | - | - | - | - | - | - | - | - | - | - | - |
| CCL21 | - | - | - | - | - | - | - | - | - | - | - | - | - | - | - | - | - | - | - | - | - | - | - | - |
| DDX58 | - | - | - | - | - | - | - | - | - | - | - | - | - | - | - | - | - | - | - | - | - | - | - | - |
| EIF2AK2 | - | - | - | - | - | - | - | - | - | - | - | - | - | - | - | - | - | - | - | - | - | - | - | - |
| IFITM3 | - | - | - | - | - | - | - | - | - | - | - | - | - | - | - | - | - | - | - | - | - | - | - | - |
| IFNA1 | - | - | - | - | - | - | - | - | - | - | - | - | - | - | - | - | - | - | - | - | - | - | - | - |
| IFNA2 | - | - | - | - | - | - | - | - | - | - | - | - | - | - | - | - | - | - | - | - | - | - | - | - |
| IFNA3 | - | - | - | - | - | - | - | - | - | - | - | - | - | - | - | - | - | - | - | - | - | - | - | - |
| IFNB | - | - | - | - | - | - | - | - | - | - | - | - | - | - | - | - | - | - | - | - | - | - | - | - |
| IFNG | - | - | - | - | - | - | - | - | - | - | - | - | - | - | - | - | - | - | - | - | - | - | - | - |
| IL1B | - | - | - | - | - | - | - | - | - | - | - | - | - | - | - | - | - | - | - | - | - | - | - | - |
| IL6 | - | - | - | - | - | - | - | - | - | - | - | - | - | - | - | - | - | - | - | - | - | - | - | - |
| IRF3 | - | - | - | - | - | - | - | - | - | - | - | - | - | - | - | - | - | - | - | - | - | - | - | - |
| LGP2 | - | - | - | - | - | - | - | - | - | - | - | - | - | - | - | - | - | - | - | - | - | - | - | - |
| MDA5 | - | - | - | - | - | - | - | - | - | - | - | - | - | - | - | - | - | - | - | - | - | - | - | - |
| MX1 | - | - | - | - | - | - | - | - | - | - | - | - | - | - | - | - | - | - | - | - | - | - | - | - |
| NFKB1 | - | - | - | - | - | - | - | - | - | - | - | - | - | - | - | - | - | - | - | - | - | - | - | - |
| NFKB2 | - | - | - | - | - | - | - | - | - | - | - | - | - | - | - | - | - | - | - | - | - | - | - | - |
| NOS2 | - | - | - | - | - | - | - | - | - | - | - | - | - | - | - | - | - | - | - | - | - | - | - | - |
| STING1 | - | - | - | - | - | - | - | - | - | - | - | - | - | - | - | - | - | - | - | - | - | - | - | - |
| TLR7 | - | - | - | - | - | - | - | - | - | - | - | - | - | - | - | - | - | - | - | - | - | - | - | - |
| TNFA | - | - | - | - | - | - | - | - | - | - | - | - | - | - | - | - | - | - | - | - | - | - | - | - |
| TRIM27.1 | - | - | - | - | - | - | - | - | - | - | - | - | - | - | - | - | - | - | - | - | - | - | - | - |
| TRIM27.2 | - | - | - | - | - | - | - | - | - | - | - | - | - | - | - | - | - | - | - | - | - | - | - | - |
| TRIM27-L | - | - | - | - | - | - | - | - | - | - | - | - | - | - | - | - | - | - | - | - | - | - | - | - |
| ZC3HAV1 | - | - | - | - | - | - | - | - | - | - | - | - | - | - | - | - | - | - | - | - | - | - | - | - |

^a^Significantly differentially expressed genes (DEGs) were identified by filtering the DESeq2 output files for gene names identical to the genes listed in the table or gene descriptions including “defensin” or “gallinacin” (for β-defensins/gallinacins) using a threshold of ≥ 10% fold change relative to control birds and an adjusted p value <0.05. “-“ indicates values below this threshold.

**Table S5. Differentially expressed genes identified by filtering DESeq2 output files from infected chickens using search terms^a^ related to glycosylation and transcription (Figure 3a)**

| Gene  Name^a^ | Lung | | | | | | | | Spleen | | | | | | | | Colon | | | | | | | |
| --- | --- | --- | --- | --- | --- | --- | --- | --- | --- | --- | --- | --- | --- | --- | --- | --- | --- | --- | --- | --- | --- | --- | --- | --- |
|  | H3 | H4 | H6 | H8 | H9 | H10 | H11 | H15 | H3 | H4 | H6 | H8 | H9 | H10 | H11 | H15 | H3 | H4 | H6 | H8 | H9 | H10 | H11 | H15 |
| ANP32B | - | - | - | - | - | - | - | - | - | - | - | - | -0,50 | - | - | - | - | - | - | - | - | - | - | - |
| ANP32E | - | - | -1,07 | -0,67 | -0,72 | -0,54 | -0,72 | -0,75 | - | - | -0,71 | -0,59 | -0,62 | -0,63 | -0,63 | -0,61 | - | - | -1,05 | -0,79 | -0,69 | -0,65 | -0,84 | -0,72 |
| ZC3H11B | - | - | - | - | 1,27 | 1,38 | 1,34 | - | - | - | 0,70 | - | 0,67 | 1,04 | 0,87 | 0,69 | - | - | 0,74 | - | 0,81 | 0,94 | 0,87 | 1,10 |
| FUT8 | - | - | 1,45 | 1,68 | 1,57 | 1,75 | 1,71 | 1,69 | - | - | 2,10 | 1,89 | 1,86 | 1,71 | 1,99 | 1,93 | 1,04 | - | 1,70 | 1,32 | 1,27 | 1,49 | 0,97 | 1,29 |
| FUT11 | - | - | -2,14 | - | -1,34 | - | - | - | - | - | -1,87 | -1,59 | -1,64 | -1,24 | -1,56 | -1,56 | - | - | -1,98 | - | - | - | -1,53 | - |
| GNPTAB | - | - | - | 0,67 | 0,53 | 0,60 | 0,64 | 0,60 | - | - | - | - | - | 0,57 | - | - | - | - | - | - | - | - | - | - |
| ST3GAL2 | - | - | - | - | - | - | - | - | - | - | - | 0,71 | 0,68 | 0,92 | 0,69 | 0,80 | - | - | - | - | - | 0,98 | - | - |
| ST3GAL4 | - | - | 1,30 | 1,21 | 1,03 | 1,27 | 1,05 | 1,34 | - | - | - | - | - | - | - | - | - | - | - | - | - | - | - | - |
| ST3GAL5 | - | - | - | - | - | - | - | - | - | - | 2,34 | 2,54 | 2,51 | 2,51 | - | 2,80 | - | - | - | - | - | - | - | - |
| ST3GAL6 | - | - | -0,57 | - | -0,61 | -0,61 | -0,61 | -0,54 | - | - | - | - | - | - | - | - | - | - | - | - | - | - | - | - |
| ST6GAL1 | - | - | -1,37 | - | -1,46 | -1,22 | -1,19 | -1,48 | - | - | -1,03 | -0,74 | -0,92 | -0,87 | -0,99 | -1,01 | -0,78 | - | -1,26 | -1,56 | -1,12 | -1,07 | -1,32 | -1,18 |
| ST6GAL2 | - | - | - | - | 2,52 | - | 2,45 | - | - | - | - | - | - | - | - | - | - | - | - | - | - | - | - | - |

^a^Significantly differentially expressed genes (DEGs) were identified by filtering the DESeq2 output files for gene names including any of the search terms: “ANP32”, ”ZC3H11”, “ST3GAL”, “ST6GAL”, “FUT”, “GNPT”, “NAGPA”, “MUC” or “CGT” using a threshold of ≥ 10% fold change relative to control birds and an adjusted p value <0.05. “-" indicates values below this threshold.

**Table S6. Differentially expressed genes identified by filtering DESeq2 output files from infected tufted ducks using search terms^a^ related to glycosylation and transcription (Figure 3b)**

| Gene  Name^a^ | Lung | | | | | | | | Spleen | | | | | | | | Colon | | | | | | | |
| --- | --- | --- | --- | --- | --- | --- | --- | --- | --- | --- | --- | --- | --- | --- | --- | --- | --- | --- | --- | --- | --- | --- | --- | --- |
|  | H3 | H4 | H6 | H8 | H9 | H10 | H11 | H15 | H3 | H4 | H6 | H8 | H9 | H10 | H11 | H15 | H3 | H4 | H6 | H8 | H9 | H10 | H11 | H15 |
| ZC3H11A | - | - | - | - | - | - | - | - | - | - | - | - | - | - | - | - | - | - | -1,42 | -1,10 | -1,13 | -1,12 | - | - |
| POFUT1 | - | - | - | -2,42 | - | - | - | - | - | - | - | - | - | - | - | - | - | - | - | - | - | - | - | - |
| FUT4 | - | - | - | - | - | - | - | - | - | -1,42 | - | - | - | - | - | - | - | - | - | - | - | - | - | - |
| FUT8 | - | - | - | - | - | - | - | - | - | 0,92 | - | 0,68 | - | - | - | - | - | - | - | - | - | - | - | - |
| FUT10 | - | - | 1.72 | - | 1,62 | - | - | - | - | - | - | - | - | - | - | - | - | - | - | - | - | - | - | - |
| ST3GAL2 | - | - | - | - | - | - | - | - | - | - | - | - | - | - | - | - | -0,91 | - | - | - | - | - | - | - |
| ST6GALNAC2 | - | - | - | - | - | - | - | - | - | - | - | - | - | - | - | - | -1,73 | - | - | - | - | - | - | - |
| ST6GALNAC4 | - | - | - | - | - | - | - | - | - | - | - | - | - | - | - | - | -1,91 | - | - | - | - | - | - | - |
| GNPTAB | - | - | - | - | - | - | - | - | 0,94 | - | - | - | 0,96 | - | - | - | - | - | - | - | - | - | - | - |
| MUC2 | - | - | - | - | 3,96 | - | - | - | 6,37 | - | - | - | - | 4,42 | - | - | - | - | - | - | - | - | - | - |

^a^Significantly differentially expressed genes (DEGs) were identified by filtering the DESeq2 output files for gene names including any of the search terms: “ANP32”, ”ZC3H11”, “ST3GAL”, “ST6GAL”, “FUT”, “GNPT”, “NAGPA”, “MUC” or “CGT” using a threshold of ≥ 10% fold change relative to control birds and an adjusted p value <0.05. “-“ indicates values below this threshold.
